# Endangered and Protected Medicinal Plants in Forest Ecosystems: Diversity, Conservation Status, Therapeutic Potential, and Management Strategies

**DOI:** 10.3390/plants15152333

**Published:** 2026-07-29

**Authors:** Ruben Budau, Mariana Florica Bei, Danut Aurel Dejeu, Manuel Alexandru Gitea, Lucian Dinca, Cristinel Constandache, Gabriel Murariu, Andrei Ioan Timofte, Daniela Gitea

**Affiliations:** 1 Department of Silviculture and Forestry Engineering, Environmental Protection Faculty, University of Oradea, General Magheru Boulevard 26, 410048 Oradea, Romania; rbudau@uoradea.ro (R.B.); andreytimofte72@gmail.com (A.I.T.); 2 Department of Food Engineering, Environmental Protection Faculty, University of Oradea, General Magheru Boulevard 26, 410048 Oradea, Romania; 3 Departament Discipline Chirurgicale, Faculty of Medicine and Pharmacy, University of Oradea, Piata 1 Decembrie 10, 410073 Oradea, Romania; ddejeu@uoradea.ro; 4 Department of Agriculture and Horticulture, Faculty of Environmental Protection, University of Oradea, General Magheru Boulevard 26, 410048 Oradea, Romania; mgitea@uoradea.ro; 5 National Institute for Research and Development in Forestry “Marin Dracea”, Eroilor 128, 077190 Voluntari, Romania; dinka.lucian@gmail.com; 6 Department of Chemistry, Physics and Environment, Faculty of Sciences and Environmental, Dunărea de Jos University Galati, Românească Street No. 47, 800008 Galati, Romania; gmurariu@ugal.ro; 7 Rexdan Research Infrastructure, “Dunarea de Jos” University of Galati, 800008 Galati, Romania; 8 General Medicine, Faculty of Medicine and Pharmacy, University of Oradea, Piata 1 Decembrie 10, 410073 Oradea, Romania; 9 Department of Pharmacy, Faculty of Medicine and Pharmacy, University of Oradea, 410048 Oradea, Romania; dgitea@uoradea.ro

**Keywords:** biodiversity conservation, ethnomedicine, habitat suitability, sustainable management, traditional knowledge

## Abstract

Medicinal plants constitute an essential component of global biodiversity and have long served as a primary source of traditional and modern therapeutic agents. Forest ecosystems harbor a significant proportion of medicinal plant diversity, providing habitats for numerous species with recognized pharmacological value. However, increasing anthropogenic pressures, including habitat destruction, overexploitation, land-use change, forest fragmentation, and climate change, have contributed to the decline of many medicinal plant populations worldwide. Consequently, a growing number of medicinal forest species are now classified as endangered, threatened, vulnerable, or protected under national and international conservation frameworks. This review synthesizes current scientific knowledge on endangered and protected medicinal plants found in forest ecosystems. A mixed-methods approach combining bibliometric assessment and qualitative literature analysis was employed to evaluate publication trends, geographical distribution, research hotspots, ecological characteristics, genetic resources, pharmacological properties, and conservation strategies related to threatened medicinal forest species. The review identified 77 medicinal plant species of conservation concern distributed across diverse forest regions worldwide, with the highest research activity originating from Asia, particularly India and China. The findings highlight the ecological importance and therapeutic potential of these species while emphasizing the critical threats posed by habitat degradation, unsustainable harvesting, and climate change. Advances in habitat suitability modeling, molecular genetics, ex situ propagation, and conservation planning have improved opportunities for species protection; however, significant knowledge gaps remain regarding population dynamics, long-term conservation effectiveness, and sustainable utilization. Strengthening integrated conservation approaches that combine habitat protection, sustainable management, scientific research, and traditional ecological knowledge is essential for safeguarding endangered medicinal forest plants and ensuring their continued contribution to biodiversity conservation, healthcare, and future drug discovery.

## 1. Introduction

Medicinal plants have played a fundamental role in human healthcare since prehistoric times and continue to represent an important source of therapeutic agents worldwide. Evidence suggests that the use of plants for medicinal purposes predates written history, with indications that even Neanderthal populations utilized plant-based remedies [1]. Despite the remarkable advances achieved in modern medicine, traditional herbal remedies remain widely used across the globe. It is estimated that approximately 75–80% of the world’s population relies, at least partially, on herbal medicines for the prevention and treatment of diseases, and the demand for plant-derived products continues to increase in both developed and developing countries [2,3,4].

Forests constitute one of the most important reservoirs of medicinal plant diversity. These ecosystems provide suitable environmental conditions for a wide range of medicinal species, including herbs, shrubs, trees, mosses, and lichens that have long been utilized in traditional medicine and are increasingly investigated for their pharmacological potential. The diversity and distribution of medicinal forest plants are influenced by numerous ecological factors, including climate, soil characteristics, altitude, and habitat structure [5,6,7,8,9]. Forest ecosystems therefore play a critical role in conserving genetic resources that support traditional healthcare systems, provide valuable sources of bioactive compounds for pharmaceutical research, and contribute to the sustainable use of medicinal plant diversity. Although thousands of medicinal plant species have been identified and utilized worldwide, the majority are still collected from natural populations rather than cultivated under controlled conditions. Only a relatively small proportion of medicinal plants are grown commercially, while wild harvesting remains the primary source of raw material for traditional medicine and the herbal industry [10]. This heavy dependence on wild resources has contributed to increasing pressure on natural populations, particularly for species characterized by slow growth, limited distribution, or specialized habitat requirements.

Currently, more than 15,000 medicinal plant species are considered threatened by extinction due to habitat loss, overexploitation, land-use change, and other anthropogenic pressures [11]. Forest medicinal plants are particularly vulnerable because many are found in highly specific ecological niches and possess biological characteristics that limit their capacity for rapid population recovery. In recent decades, deforestation, forest fragmentation, urban expansion, agricultural intensification, and climate change have accelerated the degradation of natural habitats, resulting in substantial declines in numerous medicinal plant populations worldwide. The loss of these species not only reduces biodiversity but may also lead to the disappearance of valuable bioactive compounds with potential therapeutic applications. The conservation of endangered medicinal plant species in forest ecosystems is influenced by the hydrological stability of their habitats. Climate change and land-use alterations pose severe threats to these species by shifting seasonal surface runoff and accelerating soil erosion [12,13], which can alter the specific microclimates required for their survival. Sustained protection of medicinal plant species requires proactive management and modern conservation frameworks to prevent habitat degradation due to soil erosion and torrential phenomena and ensure forest habitats are ecologically sustainable over time [14,15,16,17].

The consequences of medicinal plant decline extend beyond ecological concerns. Biodiversity loss can compromise future opportunities for drug discovery and the development of novel treatments for both existing and emerging diseases. Furthermore, uncontrolled harvesting and commercialization may reduce the availability of medicinal plants for indigenous and local communities that have relied on these resources for generations. Traditional knowledge associated with medicinal plant use is also at risk, particularly when plant populations become scarce or inaccessible [11].

For millennia, indigenous peoples and local communities have accumulated extensive knowledge regarding the identification, harvesting, preparation, and therapeutic applications of medicinal plants. This knowledge has largely been transmitted through oral traditions and cultural practices [18]. The preservation of traditional ethnobotanical knowledge, together with the conservation of medicinal plant habitats, represents an essential component of sustainable resource management. Proper documentation of traditional practices, combined with scientific validation and conservation initiatives, can contribute significantly to safeguarding both biological and cultural heritage [19]. The conservation of endangered medicinal plants requires coordinated efforts involving governments, researchers, local communities, conservation organizations, and industry stakeholders. Effective conservation strategies include the implementation of legal protection measures, sustainable harvesting protocols, habitat restoration programs, ex situ conservation approaches, public awareness campaigns, and adequate funding for biodiversity conservation initiatives [20,21]. Such integrated actions are essential to ensure the long-term survival of threatened medicinal species and the ecosystem services they provide.

Despite the increasing scientific interest in medicinal plants and biodiversity conservation, significant knowledge gaps remain regarding endangered and protected medicinal species occurring in forest ecosystems. Current research highlights several critical challenges, including insufficient assessments of conservation status, limited understanding of the impacts of climate change and habitat degradation, and inadequate information concerning effective management and restoration strategies for threatened populations [22,23]. Furthermore, many medicinal species have not yet been comprehensively evaluated regarding their population trends, genetic diversity, ecological requirements, or long-term conservation needs.

Although numerous review articles have addressed medicinal plants [24,25,26,27], ethnopharmacology, biodiversity conservation, and forest ecosystems separately [28,29,30,31], comprehensive reviews specifically focusing on endangered and protected medicinal plants from forest ecosystems remain scarce. A synthesis of current knowledge is therefore needed to improve understanding of the diversity, ecological significance, conservation status, and sustainable management of these valuable biological resources. Such information is particularly important because many modern pharmaceutical compounds originate directly or indirectly from medicinal plants, highlighting their continued relevance for healthcare and drug discovery.

Therefore, the present review aims to evaluate and synthesize the existing scientific knowledge regarding endangered and protected medicinal plants from forests. Specifically, the review focuses on (i) the diversity, distribution, and ecological characteristics of threatened medicinal forest species; (ii) environmental and anthropogenic factors affecting their populations; (iii) genetic resources and breeding characteristics relevant to conservation; (iv) pharmacological properties and therapeutic applications; (v) conservation interventions implemented for threatened taxa; and (vi) current conservation frameworks and management strategies designed to ensure the long-term survival and sustainable use of endangered medicinal forest plants. By integrating available evidence across these thematic areas, this review seeks to support future research, conservation planning, and policy development aimed at protecting these irreplaceable components of global forest biodiversity.

## 2. Results and Discussion

### 2.1. A Bibliometric Review

**Figure 1:**
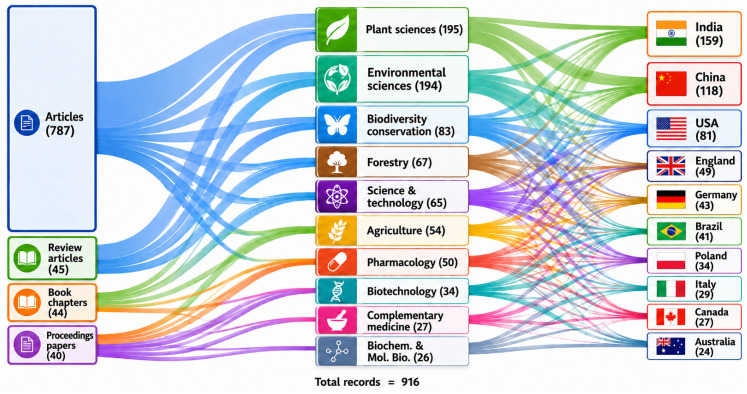
Illustrative Sankey diagram showing the distribution of publication types, major scientific areas, and leading country author clusters in the literature on endangered and protected medicinal plants from forests (n = 916 records). The width of the nodes reflects the relative contribution of each category. Because the bibliometric dataset provides aggregate counts rather than direct category linkages, the flows are schematic and intended to visualize the overall structure of the research field.

Among the 916 publications identified on this topic, the vast majority were research articles (787; 86%). These were followed by review articles, book chapters, and conference proceedings papers, which were represented in nearly equal numbers (45, 42, and 40 publications, respectively) (Figure 1).

A total of 59 research areas were identified in which the published articles can be classified. The leading fields in terms of number of publications are plant sciences (195 articles), environmental sciences–ecology (194 articles), and biodiversity conservation (83 articles). The thematic structure of the literature was therefore concentrated in plant sciences and environmental sciences–ecology, which together accounted for the largest share of publications. Additional prominent areas included forestry (67 records), science and technology (65 records), and agriculture (54 records). These results demonstrate the interdisciplinary nature of research on endangered medicinal plants, integrating botanical, ecological, conservation, and forestry perspectives.

The distribution of publication types identified in this study is consistent with findings reported in previous bibliometric analyses [32,33,34,35]. Research articles represent the dominant publication category (approximately 85–94%), followed by conference proceedings (4–12%), review papers (2–10%), and book chapters (2–3%). The predominance of original research articles indicates that the field is strongly driven by primary research, while comparatively fewer synthesis and review studies are available.

**Figure 2:**
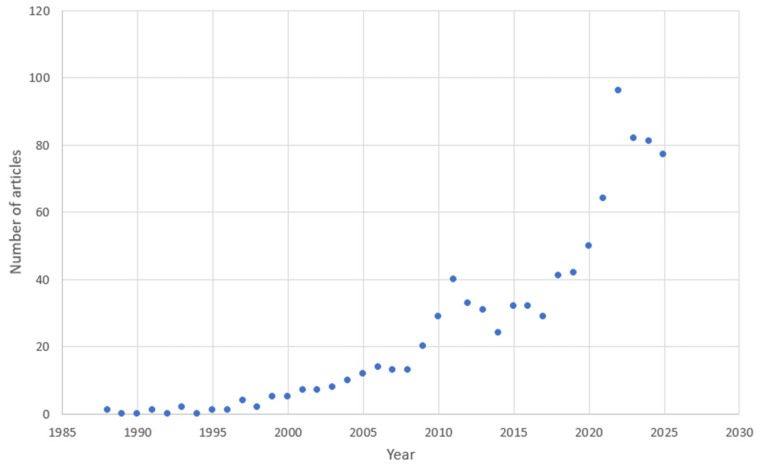
Annual distribution of articles on endangered and protected medicinal forest plants.

The number of published articles has increased over time, with the highest number recorded in 2022 (96 articles) (Figure 2). The inflection points from which the number of articles published annually began to increase at a sustained rate were the years 2010 and 2020.

The temporal pattern of scientific output mirrors trends observed in related studies [36,37,38], with a steady increase in the number of publications and particularly marked growth between 2010 and 2020. This upward trend can be attributed to the expansion of the scientific community, the overall increase in scholarly production, and the growing interest of researchers and stakeholders in the subject under investigation. Over the last few decades, the conservation and study of endangered species have become major priorities within the scientific community. This tendency is clearly reflected in research on forest plants, as demonstrated by the substantial number of publications (787), journals (371), subject categories, countries (121), and research areas (59) represented in the dataset.

**Figure 3:**
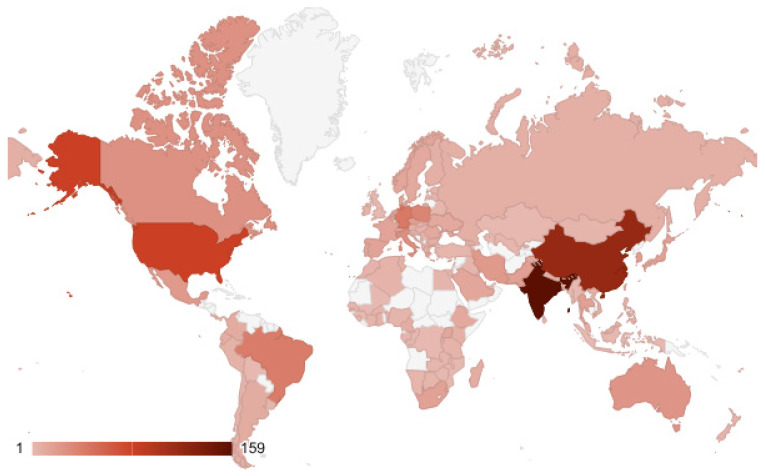
Countries with contributing authors of articles on endangered and protected medicinal forest plants.

A total of 121 countries contributed publications on this topic. India ranked first with 159 articles, followed by China with 118 articles and the United States with 81 articles (Figure 3).

Country-level analysis revealed a strong geographic concentration of research output. India (159 publications) and China (118 publications) were the leading contributors, followed by the United States (81), England (49), Germany (43), and Brazil (41). The predominance of India and China likely reflects their exceptionally rich medicinal plant diversity, extensive traditional knowledge systems, and growing scientific interest in conservation and sustainable utilization of medicinal resources. Consistent with previous bibliometric studies [39,40], countries such as the United States and China rank among the leading contributors to the literature. However, India occupies the first position in this field, likely reflecting its long-standing tradition of utilizing plant resources, including forest species, in ethnobotany and traditional medicine. Practices associated with sacred trees and the Ayurvedic system exemplify the deep cultural and historical significance of plant-based knowledge in the country.

**Figure 4:**
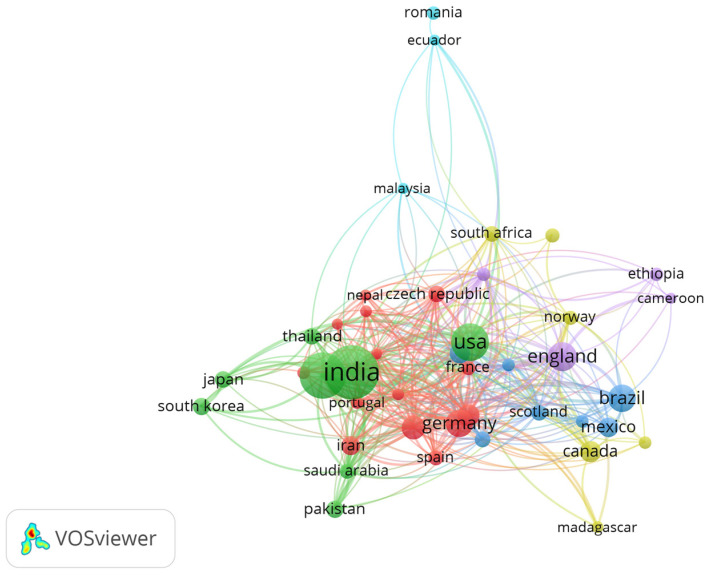
Country clusters of authors publishing on endangered and protected medicinal forest plants.

The countries of origin of authors publishing on this topic can be grouped into several clusters. The most important of these are Cluster 1, comprising countries from Europe (Czech Republic, Denmark, France, Germany, Greece, Italy, Poland, Portugal, Spain, Sweden, Switzerland, Ukraine); Cluster 2, including countries from Asia (India, Japan, Pakistan, China, Saudi Arabia, South Korea, Thailand); and Cluster 3, main consisting of countries from the Americas (Brazil, Colombia, Mexico) (Figure 4).

Regarding publishing activity, the same four major publishing houses identified in earlier studies continue to dominate the field [41,42]. In addition to the keywords used in our search—which, as expected, are among the most frequently used in the identified articles (e.g., medicinal plants, forest)—other high-impact keywords include conservation, biodiversity, ethnobotany and management (Table 1). The most frequently occurring keywords are associated with prominent research themes, including conservation, biodiversity, ethnobotany, and resource management.

**Figure 5:**
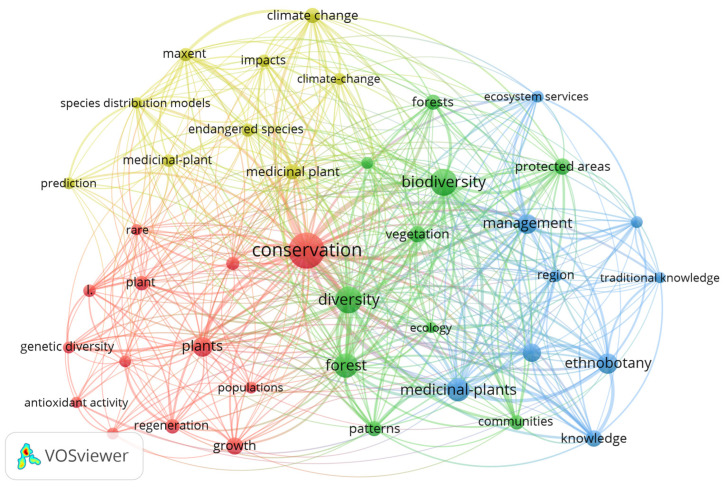
Authors’ keywords related to endangered and protected medicinal forest plants.

Based on their connections, keywords can be grouped into four clusters. Cluster 1 includes keywords related to our search (plants, endangered, rare, populations); Cluster 2 contains keywords related to forest and vegetation (forests, vegetation, protected areas); Cluster 3 includes keywords associated with ecosystems (ecosystems, biodiversity conservation, management) and Cluster 4 comprises keywords related to climate change (climate change, maxent, impacts, prediction). (Figure 5).

Overall, the Sankey visualization highlights three major characteristics of the field: (i) the overwhelming dominance of original research articles, (ii) the central role of plant science and ecological disciplines, and (iii) the leading contribution of Asian countries, particularly India and China, to research on endangered and protected medicinal plants. Together, these findings confirm the rapid development and increasing interdisciplinarity of this research area while also demonstrating broad international engagement and the growing emphasis on biodiversity conservation, sustainable resource management, and the effects of environmental change.

### 2.2. Literature Review

#### 2.2.1. Endangered and Protected Medicinal Forest Plant Species Reported in the Literature

The reviewed studies investigated a diverse range of endangered and protected medicinal plant species from forest ecosystems worldwide. Table 2 summarizes the species reported in the literature, their conservation status, geographical areas, and sources.

##### Diversity of Endangered and Protected Medicinal Plants Reported in Forest Ecosystems

The literature survey identified 77 endangered, threatened, vulnerable, rare, endemic, or legally protected medicinal plant species associated with forest ecosystems across different regions of the world (Table 2). The analyzed studies covered a wide taxonomic diversity, including trees, shrubs, herbs, orchids, and other medicinally important species. Most publications focused on species categorized as endangered or critically endangered according to national conservation assessments, the International Union for Conservation of Nature (IUCN), or other legal protection frameworks. The reviewed literature further demonstrates that medicinal plants occurring in forest ecosystems are increasingly affected by conservation challenges across multiple biogeographical regions. The predominance of endangered and critically endangered species indicates that medicinal plant resources are under considerable pressure from habitat degradation, forest fragmentation, land-use change, and unsustainable harvesting practices.

Tree species constituted a substantial proportion of the reported taxa. Among these were *Abies marocana* from Morocco [43], *Aquilaria* spp. from Malaysia [45], *Amburana cearensis* from Brazil [46], *Amphipterygium adstringens* from Mexico [48], *Boswellia serrata* [42], *Pterocarpus marsupium* [101], *Pterocarpus santalinus* [102], *Taxus wallichiana* [114], and *Warburgia salutaris* [118]. Several forest trees were recognized for both their medicinal value and their ecological importance, making them priority targets for conservation efforts. A notable pattern emerging from the literature is the dominance of tree species among threatened medicinal plants. Species such as *Aquilaria* spp. [45], *Amphipterygium adstringens* [47], *Boswellia serrata* [52], *Pterocarpus marsupium* [101], *Taxus wallichiana* [114], and *Warburgia salutaris* [118] are frequently exploited for bark, resin, wood, leaves, or other medicinally valuable plant parts. Such harvesting practices can significantly reduce regeneration capacity and accelerate population decline. Forest trees generally require long periods to reach reproductive maturity, making recovery from intensive exploitation particularly difficult.

Herbaceous medicinal species represented another major category. Frequently investigated examples included *Aconitum heterophyllum* [44], *Andrographis paniculata* [48], *Colchicum luteum* [60], *Picrorhiza kurroa* [94], *Saussurea costus* [105], *Swertia chirayita* [112], *Trillium govanianum* [116], and *Paris polyphylla* [90]. Many of these species are highly valued in traditional medicine and pharmaceutical applications. Several Himalayan medicinal herbs, including *Aconitum heterophyllum* [33], *Picrorhiza kurroa* [83], *Saussurea costus* [105], *Swertia chirayita* [112], *Taxus wallichiana* [114], and *Trillium govanianum* [116], have experienced intense collection pressure due to their high economic value in traditional and commercial medicinal systems.

Several orchid species and other rare forest understory plants have also been documented, including *Anoectochilus roxburghii* from Vietnam [49], *Cypripedium calceolus* from Poland [64], and *Gastrodia elata* from China [69]. These species are often characterized by narrow ecological requirements and limited natural populations. The reviewed studies also highlight the importance of ex situ and in vitro conservation approaches. Research on species such as *Anoectochilus roxburghii* [49], *Camptotheca acuminata* [56], *Lilium polyphyllum* [83], and *Malania oleifera* [84] demonstrates growing interest in micropropagation, tissue culture, seed conservation, and other biotechnological techniques. These methods can support the production of planting material while reducing pressure on wild populations.

##### Geographical Distribution of Research

The studies originated from Asia, Africa, Europe, North America, and South America. India emerged as the most represented country in the reviewed literature, accounting for a large number of endangered medicinal species, including *Aconitum heterophyllum*, *Andrographis paniculata*, *Boswellia serrata*, *Colchicum luteum*, *Fritillaria roylei*, *Illicium griffithii*, *Lilium polyphyllum*, *Oroxylum indicum*, *Paris polyphylla*, *Picrorhiza kurroa*, *Pterocarpus marsupium*, *Pterocarpus santalinus*, *Saussurea costus*, *Soymida febrifuga*, *Strychnos potatorum*, *Swertia chirayita*, *Taxus wallichiana*, *Trillium govanianum*, and *Zanthoxylum armatum* [44,51,52,53,70,77,83,90,94,101,102,104,105,110,112,114,119]. The large number of studies from India reflects both the country’s exceptional medicinal plant diversity and the conservation pressures affecting Himalayan and tropical forest ecosystems.

China represented another important research hotspot, with studies focusing on *Camptotheca acuminata* [56], *Cornus officinalis* [62], *Gastrodia elata* [69], *Malania oleifera* [84], *Mirabilis himalaica* [85], *Phellodendron amurense* [92], *Sinopodophyllum emodi* [108], and *Sorbus caloneura* [109].

South America also contributed significantly, particularly Brazil, where endangered medicinal trees and forest species such as *Amburana cearensis*, *Caesalpinia echinata*, *Campomanesia phaea*, *Euterpe edulis*, and *Pilocarpus microphyllus* were investigated [46,54,55,67,95,96].

Additional studies originated from Iran, South Korea, Morocco, Ethiopia, Tanzania, Nigeria, Cameroon, Benin, Burkina Faso, Greece, Poland, Costa Rica, Ecuador, Kazakhstan, Mongolia, Kenya, Mexico, Malaysia, Vietnam, and the United States, demonstrating the global relevance of medicinal plant conservation.

Another important finding is the frequent occurrence of endemic species among the reviewed taxa. Examples include *Abies marocana* in Morocco [43], *Campomanesia phaea* in Brazil [55], *Kelussia odoratissima* in Iran [80], *Conradina glabra* in the United States [61], and *Froriepia subpinnata* in the Caspian forests of Iran [72]. Endemic species typically possess narrow geographical distributions and specialized habitat requirements, which increase their vulnerability to environmental disturbances and climate-related changes.

##### Conservation Categories Reported in the Literature

Most species were classified as endangered, while others were categorized as critically endangered, threatened, vulnerable, rare, endemic, or legally protected. Critically endangered taxa included *Catamixis baccharoides* [57], *Celtis toka* [58], *Lagerstroemia minuticarpa* [82], *Lilium polyphyllum* [83], *Malania oleifera* [84], *Pleodendron costaricense* [97], *Ternstroemia cameroonensis* [115], and *Vepris onanae* [119]. Species identified as vulnerable included *Buchanania lanzan* [53] and *Saraca asoca* [104]. Several studies emphasized the role of legal protection and international conservation frameworks. The inclusion of *Panax quinquefolius* in CITES Appendix II [89], legal protection measures for *Daphne mezereum* in Poland [65], and repeated references to IUCN conservation categories illustrate the increasing integration of medicinal plant conservation into national and international policy instruments.

The reviewed studies therefore revealed a broad spectrum of conservation concerns, ranging from local rarity and restricted distribution to imminent extinction risk.

The literature further suggests that sustainable management strategies should combine habitat protection, population monitoring, cultivation programs, community participation, and regulated harvesting. For species harvested directly from natural forests, cultivation and domestication initiatives may reduce pressure on wild populations while maintaining the supply of medicinal raw materials. Such approaches appear particularly relevant for commercially valuable taxa including *Aquilaria* spp. [45], *Boswellia serrata* [52], *Paris polyphylla* [90], *Picrorhiza kurroa* [94], and *Taxus wallichiana* [114].

Overall, the evidence collected from the reviewed studies indicates that endangered and protected medicinal plants represent an important component of forest biodiversity and traditional healthcare systems. Their conservation requires coordinated actions integrating ecological research, biotechnology, sustainable utilization, legal protection, and long-term forest management policies.

#### 2.2.2. Ecological Characteristics of Endangered and Protected Medicinal Plants in Forests

The reviewed studies revealed that endangered and protected medicinal plants exhibit specific ecological requirements and are strongly influenced by habitat quality, environmental conditions, reproductive performance, and regeneration capacity. Collectively, the available evidence indicates that these species are characterized by narrow ecological niches and strong dependence on specific environmental conditions. Climatic variables, particularly temperature and precipitation, consistently emerged as key determinants of species occurrence, habitat suitability, and reproductive success [43,44,70,107,121]. Such ecological specialization contributes to the vulnerability of many medicinal plant species because even relatively small environmental changes may substantially alter habitat suitability.

Ecological niche characterization demonstrated that climatic and environmental variables are major determinants of species distribution. For the endemic Moroccan fir (*Abies marocana*), GIS-based analyses identified distinct precipitation and temperature regimes associated with the species’ natural occurrence. Germination experiments further showed that seed germination percentage and mean germination time are significantly affected by the interaction between cold stratification and temperature conditions [43]. Habitat suitability modeling of *Aconitum heterophyllum* similarly indicated that environmental and bioclimatic variables strongly influence its distribution within the Kashmir Himalaya [44]. Climate change emerged as a recurrent concern throughout the reviewed literature. Experimental evidence from *Pilocarpus microphyllus* and predictive models for *Aconitum heterophyllum* indicate that future climatic conditions may reduce habitat suitability, impair physiological performance, and increase extinction risks [44,96]. Species characterized by restricted distributions, low dispersal capacity, and specialized habitat requirements are likely to be particularly susceptible to these changes.

Advanced ecological modeling approaches have also been applied to identify suitable habitats for endangered medicinal plants. In *Silybum marianum*, machine-learning models demonstrated high predictive accuracy, with elevation, annual rainfall, temperature, and distance from roads emerging as the most influential factors affecting habitat suitability. Environmental conditions were additionally shown to influence the accumulation of silymarin, the species’ principal bioactive compound [107]. Likewise, habitat suitability analysis of *Fritillaria roylei* identified minimum temperature during the coldest period and precipitation seasonality as the primary climatic drivers of distribution. Approximately 48.47% of Himachal Pradesh was predicted to be suitable for the species, with 17.75% classified as highly suitable habitat [70]. The studies further demonstrate the growing value of ecological niche modeling and machine-learning approaches in medicinal plant conservation. These methods enable the identification of suitable habitats, assessment of species vulnerability, and selection of priority areas for restoration and reintroduction programs [44,70,107]. Such tools are increasingly important for species with fragmented populations and limited ecological information.

Population ecology studies consistently demonstrated negative effects of habitat disturbance on endangered medicinal plants. In *Colchicum luteum*, density, frequency, and abundance were significantly higher in undisturbed habitats than in areas affected by tourism, grazing, deforestation, urbanization, and transportation activities. Undisturbed sites also contained higher populations of beneficial rhizospheric microorganisms, including *Pseudomonas*, *Azotobacter*, *Rhizobium*, phosphate-solubilizing bacteria, and vesicular-arbuscular mycorrhizal fungi [60]. Similar variations in phytosociological characteristics among populations of *Fritillaria roylei* were associated with differing levels of anthropogenic pressure [70]. The importance of habitat quality is evident from studies comparing disturbed and undisturbed ecosystems. Reduced population density, abundance, and frequency in disturbed habitats were observed for *Colchicum luteum* and *Fritillaria roylei* [60,70]. Anthropogenic activities not only reduce plant populations directly but also affect associated ecological processes, including soil microbial communities that support plant nutrition, growth, and resilience. These findings emphasize the importance of maintaining intact forest ecosystems and limiting habitat degradation in areas supporting endangered medicinal plants. Reproductive ecology and regeneration capacity were identified as important factors affecting species persistence. In *Salvia majdae*, reproductive success was characterized by high pollen viability, substantial fruit set, and moderate seed production. Pollination was primarily performed by medium-sized bees, although flies and butterflies also visited flowers. Despite successful sexual reproduction, natural populations contained relatively few seedlings and young individuals [103]. Germination studies on *Abies marocana* demonstrated improved seed germination following cold stratification treatments [43], while germination in *Saussurea involucrata* increased after gibberellic acid application, although responses differed among populations [106]. Phenological observations of *Exacum bicolor* revealed a distinct annual cycle of vegetative growth, flowering, fruiting, and seed dispersal completed within seven months [66].

Regeneration constraints further limit the long-term persistence of several endangered medicinal species. Studies on the rare medicinal tree *Camptotheca acuminata* revealed that seed germination and seedling recruitment are highly sensitive to temperature and water availability. Successful germination occurred only within a narrow range of environmental conditions, whereas elevated temperatures or water stress resulted in substantial seed mortality. None of the investigated microhabitats provided conditions suitable for sustained natural regeneration, indicating that the species depends on specific forest-gap environments characterized by adequate sunlight, cool temperatures, and stable soil moisture. These regeneration limitations, together with habitat loss and overexploitation, contribute substantially to its endangered status [56]. Taken together, the ecological characteristics of endangered medicinal plants highlight the complex interactions among climate, habitat conditions, reproductive biology, population dynamics, and human disturbances. Effective conservation strategies should therefore integrate habitat protection, sustainable harvesting, ex situ propagation, restoration of degraded populations, and climate-adaptive management. Long-term ecological monitoring combined with habitat suitability modeling and reproductive biology studies will be essential for ensuring the persistence of these valuable medicinal plant resources under ongoing environmental change.

#### 2.2.3. Environmental and Anthropogenic Factors Affecting Endangered Medicinal Forest Plant Populations

The reviewed studies indicate that the persistence of endangered and protected medicinal plants in forest ecosystems is influenced by a complex interaction of anthropogenic disturbance, forest stand characteristics, climatic change, and habitat suitability and regeneration constraints operating across different spatial and temporal scales. The evidence demonstrates that these ecological and anthropogenic drivers collectively determine population viability, species distribution, and long-term conservation outcomes.

Anthropogenic disturbance has been identified as a major driver of population decline in medicinal tree species. In populations of *Amphipterygium adstringens* in tropical deciduous forests of Mexico, habitat disturbance significantly affected population dynamics. Although survival rates remained relatively stable across disturbed and protected sites, recruitment was severely limited in disturbed forests, where no seedlings were recorded during the study period. Population growth rates were consistently higher and less variable in protected forests, whereas disturbed populations exhibited growth rates below replacement levels, indicating long-term decline. Rainfall influenced demographic performance, with wetter years producing higher population growth rates, particularly in disturbed habitats where environmental variability had stronger effects [47]. Such findings emphasize the importance of maintaining intact forest ecosystems and minimizing disturbances associated with grazing, harvesting, and land-use change [122,123].

Forest stand structure also plays a critical role in determining the distribution and performance of endangered medicinal plants. Research on the endangered orchid *Cypripedium calceolus* demonstrated that its occurrence was strongly associated with proximity to silver fir trees, likely due to favorable soil moisture conditions. The probability of orchid occurrence decreased with increasing stand basal area but increased near larger trees. Morphological traits were also affected by neighboring tree species, with ramets growing near beech and sycamore exhibiting reduced leaf dimensions compared with those growing near fir. These findings highlight the importance of maintaining heterogeneous mixed forest stands for the conservation of this species [64]. The strong association between *C. calceolus* and mixed beech–fir forests further suggests that management practices promoting structural heterogeneity can improve habitat quality for sensitive medicinal species [64].

Climate change represents an increasingly important threat to endangered medicinal plants. Habitat suitability modeling for *Boswellia serrata* in eastern India predicted that approximately 20.5% of the region currently provides suitable habitat. Future climate projections suggested a reduction of about 9% in suitable habitat by 2060, with the possibility of local extinction in parts of Jharkhand, identifying climate-driven range contractions as a major conservation concern [52]. Predicted habitat contraction for *B. serrata* indicates that climate change may exacerbate existing conservation challenges by reducing the availability of suitable environments and increasing the risk of local population losses [52]. Experimental studies on *Pilocarpus microphyllus* further demonstrated that elevated temperatures, increased atmospheric CO_2_ concentrations, and water stress negatively affected growth, physiology, and biomass production, with the strongest impacts observed when future climate conditions were combined with drought [97]. Habitat suitability models for *Aconitum heterophyllum* also suggested high vulnerability to climate-driven habitat shifts [44].

However, climate change is not expected to affect all threatened medicinal species in the same way. Modeling of the critically endangered tree *Karomia gigas* in the coastal forests of Tanzania projected a substantial increase in suitable habitat by 2050 and 2070 under both low- and high-emission scenarios. Temperature annual range was identified as the principal environmental predictor, suggesting that future climatic conditions may create additional suitable habitats for this species [79]. Conversely, projections for *K. gigas* suggest that some species may benefit from future climatic conditions, potentially expanding their suitable habitat range [79]. These contrasting outcomes highlight the necessity of species-specific assessments when developing conservation and restoration plans.

Environmental variables operating at local and regional scales can strongly influence medicinal plant distributions. For *Kelussia odoratissima*, a medicinal species endemic to Iran, elevation, evaporation, and slope were identified as the most important determinants of distribution. Elevation exerted a positive effect on species occurrence, whereas higher evaporation rates and steeper slopes reduced habitat suitability [80]. The importance of environmental gradients, particularly elevation and moisture-related variables, suggests that future shifts in temperature and water availability may significantly affect habitat suitability and species persistence [80]. Integrating these environmental predictors into conservation planning can improve the identification of priority habitats and restoration sites.

Regeneration constraints represent another critical factor contributing to endangerment. Studies on the rare medicinal tree *Camptotheca acuminata* revealed that seed germination and seedling recruitment are highly sensitive to temperature and water availability. Successful germination occurred only within a narrow range of environmental conditions, while exposure to elevated temperatures or water stress resulted in substantial seed mortality. None of the investigated microhabitats provided conditions suitable for sustained natural regeneration. The species therefore depends on specific forest-gap environments characterized by adequate sunlight, cool temperatures, and stable soil moisture. Regeneration limitations, combined with habitat loss and overexploitation, contribute substantially to the endangered status of this species [56]. The sensitivity of *C. acuminata* seeds and seedlings to temperature and moisture stress demonstrates how recruitment bottlenecks can limit population recovery even where adult individuals persist [56]. Similar regeneration constraints may affect other threatened medicinal species, particularly under increasing climatic variability. Conservation programs should therefore prioritize not only the protection of mature individuals but also the maintenance of microhabitats that support successful germination and seedling establishment.

These studies indicate that the conservation of endangered and protected medicinal plants requires integrated approaches that address habitat disturbance, forest management, climate change adaptation, environmental gradients, and regeneration support. Long-term monitoring, habitat restoration, and species-specific management interventions will be essential for maintaining viable populations and preserving the ecological and medicinal value of these forest species.

#### 2.2.4. Genetic Resources and Breeding Characteristics of Endangered Medicinal Forest Species

Genetic diversity studies have provided critical information for the conservation of endangered medicinal forest plants, revealing highly variable patterns of genetic diversity and population structure that reflect differences in life history traits, breeding systems, harvesting intensity, habitat fragmentation, and historical demographic processes. Several endangered medicinal species retain considerable genetic diversity despite population decline, whereas others exhibit clear signs of genetic erosion, emphasizing that conservation strategies must be tailored to species-specific demographic and evolutionary histories.

In *Origanum compactum*, a threatened medicinal and culinary species endemic to Morocco and southern Spain, microsatellite analysis of 670 individuals from 59 populations revealed unexpectedly high genetic diversity, particularly in isolated and declining populations. Population genetic analyses identified three major genetic clusters and a strong genetic structure characterized by substantial population differentiation (Fixation index FST = 0.22) and limited gene flow (Nm = 0.88). Historical demographic analyses indicated significant population changes in 39 of the 59 populations, suggesting that habitat fragmentation and recent isolation have strongly influenced contemporary genetic patterns [87].

Similarly, genetic diversity assessments of the endangered medicinal tree *Illicium griffithii* using Random Amplified Polymorphic DNA (RAPD), Inter-simple sequence repeat (ISSR), Directed Amplification of Minisatellite-region DNA (DAMD), and Start Codon Targeted (SCoT) markers generated 250 bands, of which 98.4% were polymorphic. The species exhibited relatively low genetic differentiation among populations (Gst = 0.396; FST = 0.30) and restricted gene flow (Nm = 0.761). Bayesian clustering, principal coordinate analysis, and Unweighted Pair Group Method with Arithmetic Mean (UPGMA) dendrograms demonstrated that genetic exchange occurred primarily among geographically proximate populations, highlighting the influence of spatial isolation on population structure [66].

Research on the endangered medicinal plant *Pilocarpus microphyllus* employed Restriction-site Associated DNA (RAD) sequencing to identify 5266 neutral Single Nucleotide Polymorphisms (SNPs) in 277 individuals from the Carajás National Forest. Despite long-term harvesting pressure, populations maintained high levels of genetic diversity and formed four geographically distinct genetic clusters with substantial admixture. Geographic distance and temperature differences significantly influenced relatedness patterns. Conservation analyses suggested that at least 40 maternal plants per population should be included in germplasm collections to preserve long-term genetic diversity [95].

The endangered yew *Taxus baccata* from the Hyrcanian forests also displayed high genetic diversity when evaluated with Simple Sequence Repeats (SSR} markers. Population differentiation was relatively low (global FST = 0.044), although significant spatial genetic structure and varying degrees of inbreeding were detected. Historical analyses suggested divergence between eastern and western forest populations followed by secondary contact in central regions. Gene flow was reduced by habitat fragmentation, geographical barriers, limited regeneration, and anthropogenic disturbances [113]. Similarly to *Origanum compactum* and *Pilocarpus microphyllus*, the persistence of substantial genetic diversity demonstrates considerable resilience despite ongoing habitat disturbance [87,95,113].

In contrast, several endangered species showed evidence of severe genetic erosion. Population genomic analysis of *Conradina glabra* using 15,387 SNPs revealed low genetic diversity and the existence of two distinct genetic clusters. Clonality was frequently observed among individuals growing in close proximity. The current genetic structure was attributed to historical bottleneck events associated with habitat disturbance, emphasizing the need for ex situ conservation measures [61]. Similar concerns emerge from studies of *Caesalpinia echinata* and *Euterpe edulis*, where strong population differentiation and restricted gene flow indicate increasing vulnerability to genetic drift and local extinction [54,67]. These findings emphasize the importance of preserving multiple geographically distinct populations to maintain species-wide genetic resources. The same conclusion was obtained for three important forest tree species of Europe, Norway spruce, European beech, and Swiss stone pine, for which the use of a high variety and provenance in afforestation is recommended to ensure high adaptability of the future forests [124,125,126,127,128].

Studies of *Kirengeshoma palmata* demonstrated relatively high within-population genetic diversity and high outcrossing rates despite substantial habitat fragmentation. However, small subpopulations exhibited reduced mating opportunities and lower seed production because of mate scarcity rather than pollinator limitation. Genetic evidence suggested that local extinction of intermediate populations contributed to the observed genetic structure [75]. These findings illustrate how habitat fragmentation may reduce reproductive success even when genetic diversity remains relatively high.

Early molecular studies on *Caesalpinia echinata* using RAPD markers found that genetic variation was strongly partitioned among geographic regions and populations. Approximately 28.5% of total variation occurred between geographical groups, 29.6% among populations within groups, and 42.0% within populations. The high degree of population differentiation suggested restricted gene exchange and possible inbreeding, reflecting the long history of exploitation and habitat fragmentation affecting this species [54].

A comparable pattern was reported for *Euterpe edulis*, where Amplified Fragment Length Polymorphism (AFLP) analysis of 150 individuals from 11 populations demonstrated moderate within-population variation (57.4%) and strong population differentiation (FST = 0.426). Genetic divergence increased with geographic distance, indicating that historical fragmentation of the Atlantic Forest had significantly influenced contemporary genetic structure [67]. Restricted gene flow caused by habitat fragmentation, geographical barriers, and anthropogenic disturbances appears to be one of the principal factors limiting connectivity among endangered medicinal plant populations, increasing the risk of inbreeding and reducing long-term adaptive capacity [54,67,77,87,113].

Breeding system investigations have further clarified reproductive limitations affecting endangered medicinal species. In *Buchanania lanzan*, floral biology studies revealed a predominantly outcrossing breeding system with partial self-incompatibility. Cross-pollinated flowers produced a substantially higher fruit set (74.4%) than self-pollinated flowers (43.4%), while natural fruit production remained extremely low (3%). The limited effectiveness of natural pollinators was identified as a major constraint on population recovery, suggesting that assisted cross-pollination may be necessary for successful regeneration [53]. Similarly, the predominance of outcrossing in *Kirengeshoma palmata* promotes genetic diversity but simultaneously creates dependence on effective pollination and sufficient mate availability [75]. In fragmented populations, reduced pollinator activity or mate scarcity may substantially decrease reproductive success, even when genetic diversity remains relatively high. The recommendation for assisted cross-pollination in *Buchanania lanzan* highlights the potential role of active management interventions in species recovery programs [53,75].

Advances in genomic technologies have expanded conservation resources for endangered medicinal plants. The complete mitochondrial genome of *Fritillaria ussuriensis* was assembled using PacBio and Illumina sequencing platforms, revealing a complex mitochondrial architecture consisting of 13 circular chromosomes and 55 annotated genes. Comparative genomic analyses identified extensive repeat content, numerous RNA-editing sites, and evidence of predominantly negative selection across protein-coding genes. These genomic resources provide an important foundation for future evolutionary, conservation, and breeding studies of this endangered medicinal species [71]. Likewise, high-throughput SNP genotyping, RAD sequencing, and complete organellar genome sequencing now provide unprecedented resolution for identifying population structure, estimating effective population sizes, detecting adaptive variation, and designing germplasm collections [61,71,95]. Such genomic resources support evidence-based conservation planning and facilitate the development of breeding strategies aimed at preserving adaptive genetic variation.

The available evidence indicates that conservation strategies for endangered medicinal forest plants should integrate both in situ and ex situ approaches. Protection of genetically distinct populations, restoration of habitat connectivity, establishment of representative germplasm banks, and management practices that enhance successful cross-pollination are essential measures [53,61,75]. Conservation programs should also incorporate molecular and genomic monitoring to evaluate population trends and maintain evolutionary potential under ongoing environmental change and anthropogenic pressure [61,71,95].

#### 2.2.5. Pharmacological Properties and Therapeutic Applications of Endangered Medicinal Forest Species

A wide range of endangered and protected forest plant species possess significant medicinal value and have been extensively used in traditional healthcare systems. Scientific investigations have increasingly validated many of their ethnomedicinal applications through phytochemical and pharmacological studies. The reviewed endangered and protected medicinal forest species demonstrate substantial pharmacological diversity, reflecting the importance of forest ecosystems as reservoirs of bioactive compounds. The documented activities encompass anticancer, anti-inflammatory, antimicrobial, antioxidant, antidiabetic, hepatoprotective, and antiparasitic effects, supporting the long-standing use of these species in traditional healthcare systems [45,46,47,59,114].

Among protected tropical forest species, *Aquilaria* spp. have attracted considerable attention due to their medicinally valuable leaves, which are used to prepare agarwood tea. The leaves contain diverse bioactive compounds, including 2-(2-phenylethyl) chromones, phenolic acids, steroids, fatty acids, benzophenones, xanthonoids, flavonoids, terpenoids, and alkanes. These constituents are associated with numerous pharmacological activities, including analgesic, anti-arthritic, anti-inflammatory, anticancer, antitumor, antioxidant, antibacterial, antifungal, antidiabetic, antihistaminic, lipid-lowering, laxative, acetylcholinesterase inhibitory, and hepatoprotective effects [45]. Anticancer activity has also been reported for *Aquilaria* species, indicating that endangered forest species remain important candidates for pharmaceutical discovery and drug development [45]. Cultivation of *Aquilaria* plantations has enabled utilization of leaves for agarwood tea without requiring destruction of resin-producing trees, illustrating how conservation objectives can be integrated with medicinal resource utilization [45].

The Himalayan yew (*Taxus wallichiana*) represents one of the most important endangered medicinal tree species because of its production of taxol (paclitaxel), a diterpenoid compound with proven efficacy against several cancers, particularly breast and ovarian cancer. Leaves and bark contain substantial concentrations of this bioactive molecule, making the species a highly valuable pharmaceutical resource [114]. *Taxus wallichiana* is particularly notable because it serves as a natural source of taxol, one of the most successful plant-derived anticancer drugs used in modern oncology [114]. At the same time, commercial harvesting for taxol production has contributed to population decline, emphasizing the need for sustainable management and conservation strategies [114].

Several endangered species are recognized for their anti-inflammatory and antimicrobial activities. *Amburana cearensis*, a native tree of the Brazilian Caatinga biome, has been widely employed in traditional medicine and has become the focus of numerous pharmacological investigations. Research indicates a broad spectrum of medicinal applications, supported by an increasing number of experimental studies aimed at identifying its therapeutic compounds and biological activities [46]. Despite these promising findings, additional phytochemical characterization, pharmacological validation, toxicological assessment, and clinical studies are needed to substantiate traditional claims and facilitate the development of standardized therapeutic products [46]. Similarly, *Chamaecyparis obtusa* exhibits antimicrobial, antioxidant, anticancer, antidiabetic, antiasthmatic, anti-inflammatory, antiallergic, analgesic, and central nervous system effects. Essential oils derived from this species are also utilized in cosmetology and have demonstrated insect-repellent properties [59].

Traditional medicinal systems continue to rely on endangered forest trees. *Hagenia abyssinica* remains an important medicinal resource in Ethiopia, where different plant parts are used to treat various ailments. The flowers are particularly valued as an anthelmintic remedy, although concerns have been raised regarding toxicity when consumed in excessive amounts [73]. Likewise, *Soymida febrifuga* has a long history of use in Ayurvedic, Siddha, Unani, and folk medicine for treating malaria, diarrhea, skin diseases, and other disorders. Phytochemical investigations have identified flavonoids with antibacterial, antioxidant, and hepatoprotective activities [110]. These species remain integral components of traditional healthcare systems, particularly in rural and indigenous communities, where ethnomedicinal knowledge has frequently guided scientific investigations, facilitating the identification of pharmacologically active compounds and therapeutic applications [46,73,110,116]. Nevertheless, *Hagenia abyssinica* faces multiple anthropogenic threats, while *Soymida febrifuga* requires additional pharmacological and toxicological investigations to support future therapeutic applications [46,73,110].

Several endangered Himalayan medicinal plants have demonstrated significant therapeutic potential. *Trillium govanianum* is traditionally used to treat cancer, hypertension, arthritis, dysentery, inflammation, sepsis, reproductive disorders, giddiness, and neurasthenia [116]. Another important Himalayan species, *Saussurea costus*, is widely utilized for asthma, inflammatory diseases, ulcers, and gastrointestinal disorders. Pharmacological studies have confirmed anti-inflammatory, anti-ulcer, anticancer, and hepatoprotective properties. Major bioactive compounds include the sesquiterpene lactones costunolide, dehydrocostus lactone, and cynaropicrin [105]. The anticancer properties reported for *Saussurea costus* further demonstrate the pharmaceutical importance of endangered medicinal forest plants [105]. However, commercial harvesting of rhizomes and other medicinally valuable plant parts contributes to declining natural populations, highlighting the importance of sustainable harvesting and cultivation practices [105,116].

The critically endangered liana *Coscinium fenestratum* possesses considerable medicinal importance across South and Southeast Asia. The stem and root are rich in berberine and are traditionally employed as anti-inflammatory, antiseptic, febrifuge, and tonic agents. Therapeutic applications include treatment of wounds, ulcers, ophthalmic disorders, diabetes, fever, and general debility [63]. However, exploitation of its medicinal stem and root has intensified harvesting pressure on natural populations and increased conservation concerns [63].

Recent studies have also highlighted the medicinal significance of endangered species rich in phenolic compounds and antioxidants. *Mirabilis himalaica*, a Class I endangered medicinal plant in China, contains several bioactive phenolic acids, including rosmarinic acid, sinapic acid, salicylic acid, tyrosol, and isochlorogenic acids. These metabolites contribute to the species’ medicinal value and support its traditional applications in Tibetan medicine [85]. Likewise, *Baccaurea courtallensis*, an endangered tree species of the Western Ghats, contains terpenoids, glycosides, phenols, flavonoids, alkaloids, saponins, and tannins. Leaf extracts have demonstrated strong antioxidant activity, indicating potential pharmaceutical applications [50]. Many of these biological activities are associated with phenolic compounds, flavonoids, terpenoids, and other secondary metabolites that may provide natural therapeutic agents for managing chronic inflammatory and oxidative stress-related disorders [45,50,59,85,105,110]. Biotechnological approaches such as callus cultures in *Mirabilis himalaica* demonstrate the potential to produce valuable secondary metabolites while reducing harvesting pressure on wild populations [85].

There are many woody plants with important medicinal properties. Their habitats cover large areas in the world. Flowers, leaves, and shoots of lime (*Tilia* spp.) contain flavonoids, terpenoids, phenols, organic acids, and essential oils. Lime herbal tea is used throughout the world. *Tilia* spp. have sedative, anxiolytic, anti-inflammatory and anxiolytic properties, and are also used to treat anxiety, insomnia and respiratory diseases [129,130,131]. But the bioaccumulation of pollutants in leaves or flowers of urban trees may pose a potential health risk if used for medicinal purposes [132,133,134]. *Tilia* spp. are an important component of various protected areas, with their role in forest resilience to climate change being appreciated [135,136,137].

Different forest secondary products (honey, fruits, mushrooms) have important nutritional and medical roles [138,139,140,141].

Bushes with *Pinus mugo* and *Rhododendron hirsutum* (4070*) is a protected habitat, with an important role in the conservation of pre-alpine natural vegetation. It is threatened by human activity (cut for grazing areas and firewood). Buds, cones and resin of the dwarf mountain pine are traditionally used for cough (and other respiratory problems) treatment [4]. *Rhododendron myrtifolium* (and *R. kotschyi*) is an endemic plant in the Carpathian Mountains, often traditionally used as an anti-inflammatory, antimicrobial, and antioxidant remedy. The 4070* habitat reconstruction was the subject of some European projects [142,143]. The control methods of pine quarantine organisms (*Fusarium circinatum*, *Bursaphelenchus xylophilus*) include felling and destroying (chipping or burning) infected wood. Resistant pine genotypes are used for forest restoration/rehabilitation [144,145,146,147].

Sweet chestnut (*Castanea sativa*) has many medicinal properties. Its bark, buds, and flower extracts have cardiovascular, antioxidant, cytotoxic, antidiabetic, and nephroprotective activities [148,149,150]. The habitat of *Castanea sativa* is protected in Romania due to its high conservation value, but all the mature forests and orchards died during 1984–2013 due to the invasive pathogen *Cryphonectria parasitica*. Biological control of *C. parasitica* has improved the health status of *Ca. sativa*, and its by-products (chestnut, honey, tannin) are once again being used, both for food and medicinal purposes [151,152].

Elms (*Ulmus* spp.), especially the bark of *Ulmus rubra*, are highly valued in herbal medicine for their high mucilage content, which is useful for stomach and intestine diseases (acid reflux, ulcers, colitis, etc.), respiratory diseases (cough, bronchitis), skin problems, etc. [153]. Modern medicine values the anticancer, antioxidant, antiviral/antimicrobial, anti-inflammatory, antiallergic, and osteoblastic properties of elms [154,155,156,157]. The reconstruction of elm habitats destroyed by *Ophiostoma novo-ulmi* is based on selection of resistant/tolerant genotypes [158,159,160].

The increasing medicinal demand for endangered forest species continues to create conflicts between pharmaceutical utilization and biodiversity conservation. Commercial harvesting of bark, roots, rhizomes, stems, and other medicinally valuable plant parts often results in destructive collection practices, directly contributing to population decline and increasing extinction risk in species such as *Taxus wallichiana*, *Trillium govanianum*, *Coscinium fenestratum*, and *Hagenia abyssinica* [63,73,114,116]. Future research should therefore integrate pharmacological investigations with conservation biology, sustainable management, cultivation practices, habitat restoration, and biotechnology-based propagation methods to ensure that the medicinal benefits of endangered forest species can be utilized without compromising their long-term survival [45,46,63,73,85,105,110,114,116].

#### 2.2.6. Conservation Interventions Applied to Threatened Medicinal Forest Taxa

Studies on the conservation of endangered medicinal forest plants have employed a wide range of approaches, including habitat suitability modeling, in vitro propagation, cryopreservation, vegetative propagation, and ecological restoration. The available evidence indicates that effective conservation increasingly relies on integrating predictive ecological tools, ex situ propagation technologies, long-term germplasm preservation, and field-based restoration to enhance species recovery and maintain genetic diversity.

##### Habitat Assessment and Conservation Planning

Ecological niche modeling (ENM) has emerged as a valuable tool for identifying suitable habitats and supporting conservation planning. Adhikari et al. (2019) [82] applied ENM to the critically endangered tree *Lagerstroemia minuticarpa* in the Indian Eastern Himalayas. The species was found to occupy humid to per-humid environments, with highly suitable habitats concentrated in the Lohit and Teesta river basins. Overlaying hydroelectric project locations with habitat suitability maps revealed that 19 projects overlapped with potential habitats, indicating significant threats to species survival. The authors proposed ENM as a framework for conservation planning and reintroduction strategies at the river basin scale.

Similarly, Babar et al. (2012) [102] used the GARP, Maxent, and BIOCLIM models to predict the distribution of *Pterocarpus santalinus* (Red Sanders), an endangered medicinal tree endemic to the Eastern Ghats of India. The models identified suitable habitats mainly in the Chittoor and Kadapa districts, many of which occur outside protected areas and are exposed to substantial anthropogenic pressure. The study demonstrated the usefulness of ENM for identifying priority conservation areas.

Habitat suitability modeling was also applied to *Cornus officinalis*, a medicinal species experiencing habitat degradation due to increasing market demand and forest disturbance. Cao et al. (2016) [62] combined Maxent modeling with fuzzy logic analyses and identified six key environmental variables influencing both habitat suitability and medicinal compound accumulation. Highly suitable habitats were concentrated in east-central China, highlighting regions that should receive priority for habitat rehabilitation and resource conservation.

These studies demonstrate that habitat suitability modeling and spatial conservation analyses provide robust tools for identifying priority areas for protection, restoration, and species reintroduction. When integrated with information on habitat quality, protected-area networks, land-use change, and future climate scenarios, these approaches enable conservation managers to identify populations at greatest risk, evaluate anthropogenic threats, optimize resource allocation, and support reintroduction planning, making them increasingly valuable under conditions of rapid land-use change and climate variability [62,82,102].

##### In Vitro Propagation and Micropropagation

Ex situ conservation through tissue culture has proven highly effective for numerous endangered medicinal forest species. For *Juniperus drupacea*, an endangered medicinal conifer native to southern Greece, Ioannidis et al. (2023) [78] established the first in vitro propagation protocol using shoot-tip explants. The highest bud proliferation was achieved on Driver and Kuniyaki Walnut medium supplemented with thidiazuron or meta-topolin. Although rooting remained difficult, the study provided an important foundation for future conservation efforts.

Budău et al. (2023) [161], in their study on in vitro propagation of several valuable selections of *Robinia pseudoacacia* L. as a fast and sustainable source for wood production but also for its flowers with medicinal properties, aimed to establish an alternative to the traditional propagation of a number of selections of *Robinia pseudoacacia* L. by developing an in vitro culture protocol. The subject of this study is of great importance and reflects a continuing concern of the sustainable research program of the Faculty of Environmental Protection of the University of Oradea.

Bayarmaa et al. (2018) [120] developed a somatic embryogenesis protocol for the endangered Mongolian medicinal plant *Zygophyllum potaninii*. Embryogenic calli and somatic embryos were successfully induced, and more than half of the regenerated plants survived acclimatization, demonstrating the feasibility of large-scale propagation.

For *Ilex guayusa*, Carvalho et al. (2021) [76] established an in vitro propagation system based on stem explants. The protocol achieved effective shoot development, spontaneous rooting in most shoots, and successful acclimatization, offering a sustainable alternative to harvesting from natural populations.

Demétrio et al. (2021) [55] optimized tissue culture conditions for *Campomanesia phaea*, a threatened medicinal species of the Brazilian Atlantic Forest. Reduced salt concentrations and acidic pH improved growth, while nodal explants cultured with benzylaminopurine produced the best shoot regeneration. Acclimatized plants exhibited a survival rate of 94.4%.

Monalisa et al. (2024) [51] developed an efficient regeneration system for the threatened medicinal herb *Blepharispermum subsessile*. Cotyledonary node explants produced multiple shoots on media containing meta-topolin and indole-3-acetic acid. Rooted plantlets were successfully acclimatized, and molecular analyses confirmed genetic fidelity between regenerated plants and the source material.

Micropropagation has also been successfully employed for *Decalepis arayalpathra*, an endangered medicinal species of the Western Ghats. Gangaprasad et al. (2005) [66] achieved shoot multiplication through nodal cultures and subsequently reintroduced regenerated plants into a reserve forest, where 84% survived after two years.

Guo et al. (2023) [109] established protocols for shoot proliferation, adventitious regeneration, rooting, and callus suspension culture for the endangered species *Sorbus caloneura*, providing valuable tools for germplasm conservation and future breeding.

Jani et al. (2015) [100] reported a highly reproducible regeneration system for *Psoralea corylifolia*, obtaining direct adventitious shoot regeneration from root explants and achieving 100% survival following transfer to soil. Kagithoju et al. (2013) [111] successfully achieved embryo culture to conserve the endangered medicinal forest tree *Strychnos potatorum*, obtaining complete embryo germination and acclimatized plants with survival rates of 65–75%.

Nowakowska et al. (2023) [65] developed a micropropagation protocol for the protected medicinal shrub *Daphne mezereum*, in which meta-topolin promoted the highest shoot proliferation and indole-3-butyric acid stimulated efficient root formation. Micropropagation was also successfully applied to *Khaya grandifoliola*, an endangered medicinal and timber tree from West Africa, with Okere and Adegeye (2011) [81] obtaining optimal shoot and root development using benzylaminopurine and naphthalene acetic acid.

Purohit et al. (2017) [119] established the first micropropagation protocol for *Zanthoxylum armatum*, achieving efficient shoot proliferation, complete rooting, and 75% survival during acclimatization and confirming genetic stability among regenerated plants through molecular analyses.

Collectively, these studies demonstrate the versatility of in vitro propagation methods for producing large numbers of genetically uniform plants from limited source material, making them particularly valuable for species with poor seed viability, low natural regeneration rates, fragmented populations, or reproductive barriers. Somatic embryogenesis and embryo rescue further expand conservation opportunities, as illustrated by the successful regeneration of *Zygophyllum potaninii* [120] and embryo culture of *Strychnos potatorum* [111].

##### Cryopreservation and Long-Term Germplasm Conservation

Cryopreservation has been investigated as a strategy for long-term conservation of endangered medicinal plants. Kioko et al. (1999) [118] demonstrated that seeds of *Warburgia salutaris* could tolerate substantial dehydration and remain viable after cryostorage in liquid nitrogen. Although post-thaw germination reached only 30%, the recovered seedlings appeared normal.

Parasher et al. (2023) [98] developed cryopreservation protocols for the highly endangered medicinal species *Podophyllum hexandrum*, and the V cryo-plate technique achieved 90% regrowth of zygotic embryos, significantly outperforming conventional vitrification methods. Regenerated embryos successfully developed into complete plantlets. These studies indicate that ultra-low-temperature storage can preserve valuable genetic resources while minimizing maintenance costs and the risks associated with living collections, and the high recovery rates obtained using modern cryo-plate techniques suggest considerable potential for wider application to other threatened medicinal taxa [98,118].

##### Vegetative Propagation and Restoration

Vegetative propagation and ecological restoration represent additional components of integrated conservation strategies. Tchoundjeu et al. (2004) [91] demonstrated that *Pausinystalia johimbe*, an economically important medicinal tree threatened by unsustainable bark harvesting, could be propagated effectively using juvenile leafy stem cuttings. Rooting was enhanced by auxin treatments, particularly indole-3-butyric acid.

For the critically endangered tree *Pleodendron costaricense*, Pillco Huarcaya et al. (2022) [97] successfully germinated seeds and produced 59 saplings suitable for restoration planting while identifying potential natural seed dispersers and emphasizing the need to protect seedlings from predation.

The successful reintroduction of regenerated *Decalepis arayalpathra* plants into natural forest habitats with high long-term survival [66] and the restoration program developed for *Pleodendron costaricense* [97] demonstrate that propagation technologies achieve their greatest conservation value when integrated with habitat protection and restoration initiatives. Likewise, vegetative propagation methods provide practical and cost-effective alternatives for community-based conservation programs by reducing harvesting pressure on wild populations while supporting sustainable utilization, as demonstrated for *Pausinystalia johimbe* [91].

The collective evidence indicates that no single conservation strategy is sufficient for endangered medicinal forest species. Habitat suitability assessment, in vitro propagation, cryopreservation, restoration planting, vegetative propagation, and sustainable utilization programs function as complementary components of comprehensive conservation frameworks. Their integration enhances species recovery, preserves genetic diversity, improves conservation planning, and contributes to the long-term availability of medicinal plant resources in forest ecosystems.

#### 2.2.7. Conservation Frameworks and Management Strategies for Endangered Medicinal Forest Species

The reviewed studies demonstrated that effective conservation of endangered medicinal forest plants requires integrating ecological assessments, population monitoring, sustainable utilization practices, stakeholder participation, and both in situ and ex situ measures. The evidence indicates that successful conservation depends on combining ecological, biological, socio-economic, and policy dimensions rather than relying on a single intervention.

A structured framework for conservation planning was proposed for endemic medicinal tree species of the genus *Boswellia* on Socotra Island. The approach combined ecological niche characterization, population structure analysis, regeneration assessment, and the application of IUCN Red List criteria to establish conservation priorities. The study revealed considerable differences among species in abundance, distribution, and vulnerability. Ground-rooted species such as *Boswellia ameero*, *B. elongata*, and *B. socotrana* exhibited broad distributions but showed poor regeneration, whereas cliff-rooted species such as *B. nana* and *B. bullata* were identified as highly vulnerable because of their restricted populations and limited distribution ranges. Species-specific conservation actions were therefore recommended according to their ecological characteristics and threat levels [162].

Strengthening population recruitment represents another important component of conservation planning. Seed-based conservation strategies were investigated for *Swertia chirayita*, a critically endangered medicinal herb of the Himalayas. Pre-sowing treatments significantly enhanced seedling emergence and vigor. Gibberellic acid (GA_3_) treatment increased seedling emergence from 6% in untreated controls to 69%, while also accelerating emergence rates across seed sources. Morphological evaluation identified collar diameter as a reliable indicator of seedling vigor, providing a practical criterion for selecting planting material for conservation and restoration programs. The successful enhancement of germination and seedling vigor illustrates how relatively simple propagation techniques can contribute to both ex situ conservation and population recovery initiatives, particularly for species characterized by poor natural regeneration or fragmented populations [112].

Studies on *Panax quinquefolius* highlighted the role of regulatory frameworks in medicinal plant conservation. Following its inclusion in CITES Appendix II, harvesting and international trade became subject to legal restrictions. Stakeholder surveys indicated broad support for conserving wild ginseng populations, although many participants questioned the effectiveness of top-down regulatory measures alone. Respondents emphasized the importance of traditional stewardship practices, local knowledge, and community involvement and advocated complementary conservation initiatives such as government-supported planting programs. These findings indicate that regulatory protection alone may not guarantee long-term conservation success and that community-supported conservation programs may improve compliance, strengthen monitoring capacity, and promote sustainable resource use while complementing formal legal frameworks [89].

Climate change and habitat alteration further reinforce the need for adaptive conservation strategies. Ex situ conservation through species introduction was evaluated for *Prunus ulmifolia*, a rare medicinal and ornamental tree with a naturally restricted distribution in Central Asia. Evidence from botanical gardens, arboreta, and experimental plantations demonstrated successful establishment across a wide range of climatic conditions. Distribution modeling further suggested that the species possesses considerable adaptive capacity and may shift its range northward under future climate scenarios, supporting the value of assisted cultivation and introduction programs as conservation tools. These findings suggest that ex situ collections and assisted establishment programs can serve as valuable safeguards against future environmental change, particularly for species whose natural habitats are expected to undergo substantial climatic shifts [99].

The reviewed literature also underscores the importance of reducing pressure on wild populations through cultivation and propagation. For the endangered medicinal tree *Oroxylum indicum*, habitat degradation and overexploitation were identified as major threats to natural populations. The literature highlighted the potential of tissue culture-based techniques, including embryo culture, meristem culture, and somatic embryogenesis, for large-scale propagation and germplasm conservation. These methods were proposed as essential components of future conservation programs aimed at reducing harvesting pressure on wild populations [88].

Similarly, resource assessments of *Sinopodophyllum emodi* revealed that excessive harvesting of underground plant parts for podophyllotoxin extraction represented the primary cause of population decline. The species was found to be strongly influenced by environmental factors such as altitude, temperature, soil conditions, moisture availability, and light exposure. Conservation recommendations included stricter regulation of wild collection together with the expansion of cultivation and commercial production systems to reduce pressure on natural populations [108]. Both *Oroxylum indicum* and *Sinopodophyllum emodi* illustrate how growing commercial demand can accelerate population declines when harvesting remains dependent on natural stands, whereas the development of large-scale propagation systems and cultivation programs offers a practical pathway to maintain medicinal resource availability while supporting species conservation [88,108].

**Figure 6:**
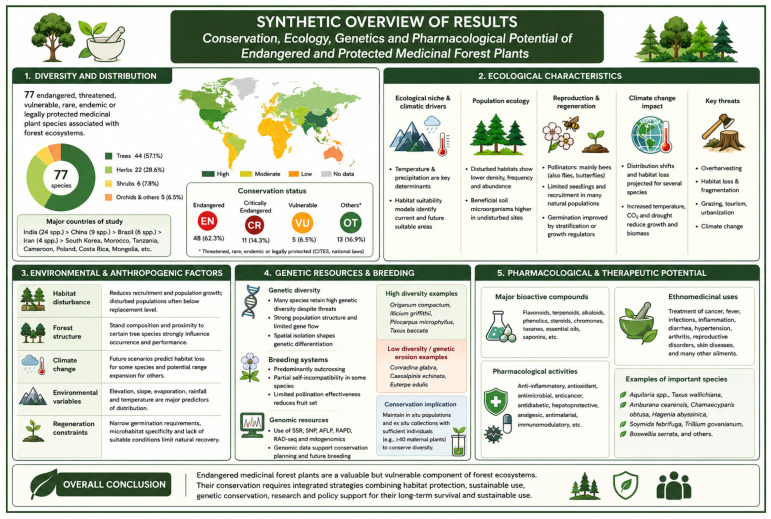
Synthetic overview of the main results obtained from the review of endangered and protected medicinal forest plants.

Figure 6 provides a synthetic overview of the principal findings regarding species diversity, ecological characteristics, conservation challenges, genetic resources, and pharmacological importance of threatened medicinal plants in forest ecosystems. The analysis identified 77 endangered, threatened, vulnerable, rare, endemic, or legally protected medicinal plant species. Trees represented the dominant growth form, followed by herbaceous species (including orchids) and shrubs. The geographical distribution highlights the strong contribution of Asian regions, particularly India and China, to research on medicinal plant conservation.

The ecological synthesis shows that the survival of these species is strongly influenced by climatic variables, habitat suitability, forest structure, regeneration capacity, and anthropogenic pressures such as habitat loss, overharvesting, fragmentation, and climate change. Genetic studies demonstrate that many species maintain valuable genetic diversity but are increasingly affected by isolation and population decline, emphasizing the need for both in situ and ex situ conservation strategies.

The pharmacological synthesis highlights the therapeutic potential of endangered medicinal plants, including bioactive compounds with antioxidant, anti-inflammatory, antimicrobial, anticancer, and other medicinal properties. Figure 6 further illustrates that conservation programs are most effective when they combine population assessment, propagation and cultivation technologies, habitat management, stakeholder participation, sustainable use strategies, genetic resource preservation, and continued scientific research. Such integrated approaches can simultaneously address biological threats, market pressures, and long-term environmental change, thereby improving the prospects for the persistence and sustainable utilization of threatened medicinal forest species.

### 2.3. Research Gaps and Future Directions

Despite the growing body of literature on endangered and protected medicinal plants from forest ecosystems, several important knowledge gaps remain. Addressing these limitations is essential for developing effective conservation strategies and ensuring the sustainable utilization of medicinal plant resources.

#### 2.3.1. Insufficient Population and Conservation Assessments

Although numerous medicinal plant species have been classified as endangered, threatened, or vulnerable, many forest medicinal plants have not been comprehensively evaluated regarding their conservation status. Population size, demographic structure, regeneration capacity, and long-term population trends remain poorly documented for many species, particularly in biodiversity-rich regions of Asia, Africa, and South America. Future studies should prioritize standardized population monitoring programs and periodic reassessments using IUCN criteria to improve conservation planning.

#### 2.3.2. Limited Understanding of Climate Change Impacts

Climate change is increasingly recognized as a major threat to forest medicinal plants, yet its long-term effects remain insufficiently understood. Most existing studies rely on habitat suitability models, while relatively few combine ecological monitoring, physiological measurements, and experimental approaches. Future research should investigate species-specific responses to changing temperature, precipitation patterns, drought frequency, and extreme climatic events. Integrating climate projections with demographic and genetic data will improve predictions of future species vulnerability.

#### 2.3.3. Knowledge Gaps in Genetic Diversity and Adaptive Potential

Genetic studies have been conducted for only a limited number of endangered medicinal species. Consequently, the extent of genetic erosion, inbreeding, and adaptive capacity remains unknown for many taxa. Future research should employ advanced genomic tools, including whole-genome sequencing, SNP analyses, landscape genomics, and environmental DNA approaches, to identify genetically important populations and support conservation breeding programs. Greater attention should also be given to the relationship between genetic diversity and medicinal compound production.

#### 2.3.4. Inadequate Information on Reproductive Ecology and Regeneration

Many endangered medicinal plants exhibit poor natural regeneration, yet the mechanisms underlying recruitment failure are often poorly understood. Limited information exists regarding pollination biology, seed dispersal, germination ecology, soil microbiome interactions, and seedling establishment. Long-term ecological studies are required to identify critical life-history bottlenecks and develop effective restoration protocols. Research on mutualistic interactions involving pollinators, mycorrhizal fungi, and other soil microorganisms should receive particular attention.

#### 2.3.5. Underrepresentation of Forest Ecosystem Processes

Most studies focus on individual species, while broader ecosystem-level processes receive less attention. The influence of forest structure, habitat connectivity, disturbance regimes, hydrological processes, and ecosystem functioning on medicinal plant populations remains insufficiently explored. Future investigations should adopt landscape-scale and ecosystem-based approaches to better understand how forest management practices affect medicinal plant conservation.

#### 2.3.6. Limited Evaluation of Sustainable Harvesting Practices

Overharvesting remains one of the primary causes of medicinal plant decline. However, quantitative studies evaluating sustainable harvesting thresholds, recovery rates, and harvesting impacts on population viability are scarce. Future research should develop evidence-based harvesting guidelines that balance conservation objectives with local livelihood needs. Comparative studies assessing wild harvesting, cultivation, and agroforestry production systems would contribute to sustainable resource management.

#### 2.3.7. Need for Improved Ex Situ Conservation Strategies

Although tissue culture, micropropagation, seed banking, and cryopreservation have been successfully applied to some endangered species, many medicinal plants remain underrepresented in ex situ conservation programs. Future efforts should focus on optimizing propagation protocols, establishing regional germplasm repositories, and integrating ex situ collections with in situ conservation initiatives. The long-term genetic representativeness of ex situ collections should also be evaluated.

#### 2.3.8. Insufficient Integration of Traditional Knowledge and Scientific Research

Traditional ecological and ethnobotanical knowledge continues to provide valuable information regarding medicinal plant use, harvesting practices, and conservation. However, many indigenous knowledge systems remain poorly documented and are increasingly threatened by cultural change. Future research should promote participatory approaches involving indigenous peoples and local communities while ensuring equitable benefit-sharing and protection of intellectual property rights. Integrating traditional knowledge with modern ecological and pharmacological research may generate innovative conservation solutions.

#### 2.3.9. Limited Pharmacological and Phytochemical Investigation

Although numerous endangered medicinal plants are traditionally used for therapeutic purposes, only a fraction have undergone rigorous phytochemical and pharmacological evaluation. Future studies should focus on identifying bioactive compounds, elucidating mechanisms of action, assessing toxicity profiles, and validating traditional medicinal applications through clinical research. Such investigations may simultaneously support biodiversity conservation and pharmaceutical innovation.

#### 2.3.10. Strengthening Conservation Policies and International Collaboration

Conservation outcomes are often constrained by fragmented legislation, insufficient funding, and weak implementation of management plans. Future research should evaluate the effectiveness of existing conservation policies, protected area networks, and species recovery programs. Greater international collaboration, data sharing, and interdisciplinary research frameworks will be essential for addressing transboundary conservation challenges and achieving global biodiversity targets.

#### 2.3.11. Future Perspectives

Future conservation of endangered medicinal forest plants will require an integrated approach combining ecological monitoring, genomic technologies, habitat restoration, sustainable harvesting, ex situ conservation, climate adaptation planning, and community participation. Advances in remote sensing, geographic information systems, artificial intelligence, and environmental DNA analysis offer promising opportunities for improving species monitoring and conservation decision-making. Strengthening the link between biodiversity conservation, traditional knowledge, and evidence-based management will be essential for ensuring the long-term persistence and sustainable use of endangered medicinal plant diversity.

## 3. Materials and Methods

This review employed a mixed-methods approach integrating systematic bibliometric analysis with qualitative thematic synthesis to examine the scientific literature concerning endangered and protected medicinal plants occurring in forest ecosystems. The methodological framework was designed to identify publication trends, research hotspots, geographic patterns, and major thematic developments in the field while ensuring transparency and reproducibility.

### 3.1. Bibliometric Assessment

#### 3.1.1. Literature Search Strategy

A systematic literature search was conducted using two internationally recognized scientific databases: Scopus and the Science Citation Index Expanded (SCI-Expanded) available through the Web of Science (WoS) platform. These databases were selected because of their extensive coverage of peer-reviewed literature in forestry, ecology, biodiversity conservation, ethnobotany, environmental sciences, and medicinal plant research.

The search focused on publications addressing endangered and protected medicinal plant species associated with forest ecosystems. Core search terms included combinations of “endangered medicinal plants”, “protected medicinal plants”, and “forest plants”. To broaden the scope and capture related studies, additional keywords included medicinal species, rare medicinal plants, threatened medicinal plants, vulnerable species, forest medicinal resources, forest biodiversity, plant conservation, in situ conservation, ex situ conservation, ethnobotany, non-timber forest products, and sustainable harvesting.

Boolean operators (AND, OR) and wildcard symbols were applied to retrieve variations in terminology and maximize search coverage. All retrieved records were screened and refined according to the Preferred Reporting Items for Systematic Reviews and Meta-Analyses (PRISMA) framework to ensure methodological rigor and reproducibility [163].

#### 3.1.2. Search Syntax

To facilitate reproducibility, the search strings used in each database are presented below, with minor adaptations according to database-specific requirements.

Scopus (Advanced Search; TITLE-ABS-KEY fields):

TITLE-ABS-KEY (“endangered medicinal plant*” OR “threatened medicinal plant*” OR “rare medicinal plant*” OR “protected medicinal plant*”) AND (forest* OR woodland* OR “forest ecosystem*” OR “forest biodiversity”) OR

TITLE-ABS-KEY (“medicinal species” OR ethnobotan* OR “non-timber forest product*”) AND (conservation OR protection OR preservation).

Web of Science—SCI-Expanded (Topic Search; TS):

TS = (“endangered medicinal plant*” OR “threatened medicinal plant*” OR “rare medicinal plant*” OR “protected medicinal plant*”) AND (forest* OR woodland* OR “forest ecosystem*” OR “forest biodiversity”) OR

TS = (“medicinal species” OR ethnobotan* OR “non-timber forest product*”) AND (conservation OR protection OR preservation).

Wildcards were used to capture plural forms and lexical variations, while equivalent logical operators were maintained across both databases.

#### 3.1.3. Search Limits and Eligibility

No temporal restrictions were imposed, allowing the inclusion of all relevant literature indexed up to the date of the search. Only peer-reviewed research articles and review papers published in English were considered eligible. Editorials, conference abstracts, proceedings papers, theses, dissertations, book chapters, and other non-peer-reviewed publications were excluded.

#### 3.1.4. Data Cleaning and De-Duplication

Duplicate records were removed through a two-stage procedure. First, automated matching based on Digital Object Identifiers (DOIs) and exact title correspondence was performed using Microsoft Excel. Second, manual verification was conducted to resolve inconsistencies associated with missing DOIs, variations in author names, or minor title differences.

Metadata quality was subsequently verified, including publication titles, abstracts, author names, institutional affiliations, publication years, and journal titles. Standardization procedures were applied to harmonize author and institutional names, thereby improving consistency in subsequent bibliometric analyses.

#### 3.1.5. Selection Criteria and Screening Procedure

Study selection followed a two-stage screening process involving title/abstract evaluation followed by full-text assessment.

Inclusion criteria:

- Peer-reviewed research articles or review papers;
- Published in English;
- Explicit focus on endangered, threatened, rare, vulnerable, or legally protected medicinal plant species associated with forest ecosystems;
- Studies addressing conservation, ecology, distribution, sustainable use, ethnobotanical importance, management, or protection measures;
- Availability of complete bibliographic metadata.

Exclusion criteria:

- Non-peer-reviewed publications;
- Studies not primarily focused on medicinal forest plant species;
- Articles in which conservation, protection, management, or ecological aspects of medicinal plants were not a central research objective;
- Incomplete abstracts or inaccessible full texts;
- Insufficient methodological information.

Two independent reviewers conducted title and abstract screening. Publications considered potentially relevant by either reviewer were advanced to full-text evaluation. Disagreements were resolved through discussion and, when necessary, consultation with a third reviewer.

Reasons for exclusion during full-text assessment were categorized as (A) out of scope; (B) non-peer-reviewed publication; (C) insufficient data; (D) inaccessible text; or (E) inadequate methodology.

#### 3.1.6. Final Dataset and Bibliometric Variables

**Figure 7:**
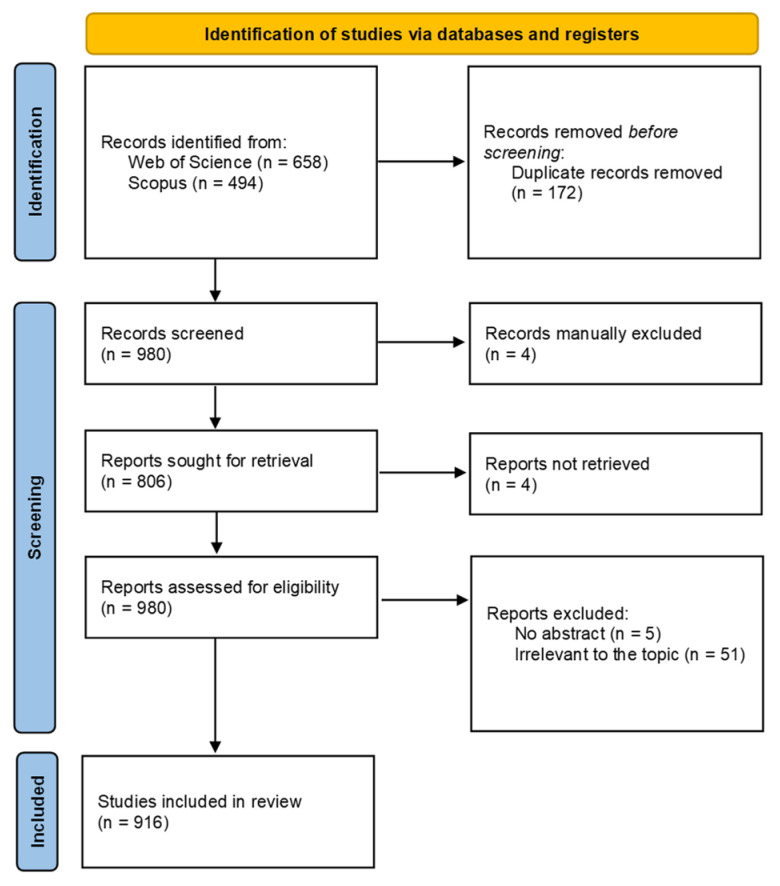
Selection process of the eligible reports based on the PRISMA 2020 flow diagram [169].

Following the screening process, the final dataset consisted of eligible publications addressing endangered and protected medicinal forest plants (Figure 7). Bibliometric indicators were analyzed across nine dimensions: publication type, research discipline, temporal publication trends, geographic distribution, authorship patterns, institutional affiliations, journal sources, publishing outlets, and keyword occurrence.

Data processing and statistical analyses were conducted using Web of Science Core Collection, Scopus, Microsoft Excel, and Geochart [164,165,166,167]. Visualization of co-authorship networks, co-citation structures, and keyword co-occurrence patterns was performed using VOSviewer (v.1.6.20) [168].

### 3.2. Qualitative Content Analysis

In parallel with the bibliometric assessment, a qualitative content analysis was conducted on the selected publications to identify dominant research themes, conceptual approaches, and methodological trends related to endangered and protected medicinal plants in forest ecosystems.

The full texts were systematically reviewed and classified into seven thematic clusters: endangered and protected medicinal forest plant species reported in the literature; ecological characteristics of endangered and protected medicinal plants in forests; environmental and anthropogenic factors affecting endangered medicinal forest plant populations; genetic resources and breeding characteristics of endangered medicinal forest species; pharmacological properties and therapeutic applications of endangered medicinal forest species; conservation interventions applied to threatened medicinal forest taxa; conservation frameworks and management strategies for endangered medicinal forest species.

**Figure 8:**
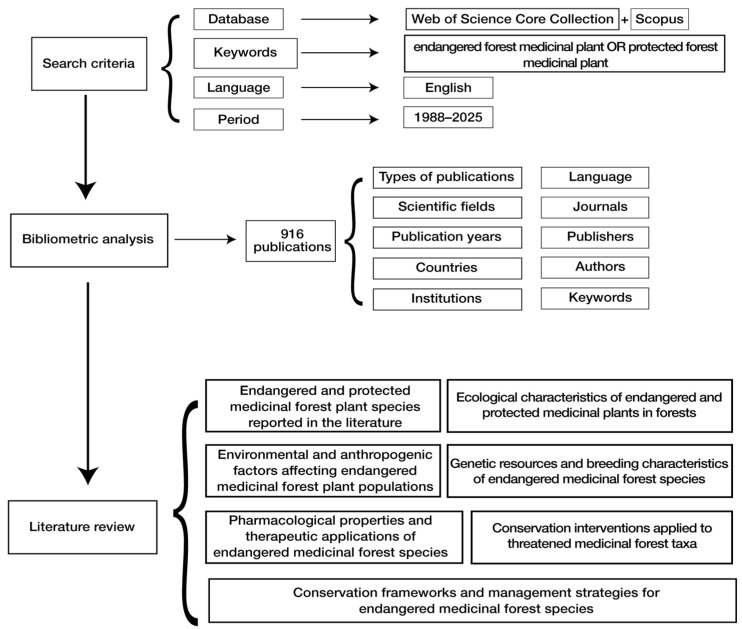
Schematic presentation of the workflow used in our research.

Thematic classification was performed through iterative coding and cross-validation among reviewers. These thematic categories provide the conceptual foundation for the Results and Discussion sections and are summarized schematically in Figure 8.

## 4. Conclusions

Medicinal plants occurring in forest ecosystems represent an invaluable component of global biodiversity and continue to play a fundamental role in traditional healthcare systems, modern medicine, and pharmaceutical development. This review synthesized current knowledge on endangered and protected medicinal plants from forests, highlighting their diversity, ecological significance, pharmacological value, conservation status, and management challenges.

The literature reveals that numerous medicinal plant species are currently threatened by habitat loss, forest fragmentation, overharvesting, climate change, land-use transformation, and declining regeneration capacity. The reviewed studies documented a wide range of endangered, vulnerable, rare, endemic, and legally protected species distributed across diverse forest ecosystems worldwide, with particularly high research activity concentrated in Asia, especially India and China. Ecological investigations demonstrated that many medicinal plants possess narrow habitat requirements and are highly sensitive to environmental changes, while genetic studies revealed varying levels of genetic diversity, population fragmentation, and reproductive constraints that influence their long-term persistence.

Pharmacological research has confirmed the significant therapeutic potential of many endangered medicinal species, including their anti-inflammatory, antimicrobial, antioxidant, anticancer, and other bioactive properties. However, the increasing commercial demand for medicinal plant resources continues to intensify harvesting pressure on wild populations, further exacerbating conservation concerns. The loss of these species would not only diminish forest biodiversity but could also eliminate valuable genetic resources and bioactive compounds with future medicinal applications.

A wide range of conservation interventions have been developed, including habitat suitability modeling, in situ protection, ecological restoration, micropropagation, cryopreservation, seed banking, and conservation-oriented breeding programs. Nevertheless, important knowledge gaps remain regarding species population dynamics, climate change responses, reproductive ecology, genetic adaptation, sustainable harvesting thresholds, and the effectiveness of existing conservation measures. These limitations highlight the need for more integrated and interdisciplinary research approaches.

The long-term conservation of endangered medicinal forest plants requires coordinated efforts involving scientists, policymakers, forest managers, local communities, and the pharmaceutical sector. Future strategies should integrate biodiversity conservation, sustainable resource use, advanced genomic technologies, habitat restoration, climate adaptation planning, and the preservation of traditional ethnobotanical knowledge. Strengthening international collaboration, improving conservation policies, and promoting evidence-based management practices will be essential for safeguarding these irreplaceable biological resources.

Ultimately, protecting endangered medicinal plants in forest ecosystems is not only a matter of species conservation but also an investment in ecosystem resilience, cultural heritage, human health, and future opportunities for scientific and pharmaceutical innovation. Ensuring their survival will contribute significantly to global biodiversity conservation and the sustainable use of natural resources for generations to come.

## Figures and Tables

**Table 1:** Most frequently used keywords in articles on endangered and protected medicinal forest plants.

Crt. No.	Keyword	Occurrences	Total Link Strength
1	conservation	144	349
2	biodiversity	83	215
3	medicinal plants	82	220
4	diversity	81	204
5	forest	68	167
6	management	45	132
7	ethnobotany	46	115
8	protected areas	30	99
9	knowledge	31	86
10	climate change	26	77
11	vegetation	31	73
12	maxent	20	67
13	patterns	27	67
14	communities	23	63

**Table 2:** Endangered and protected medicinal forest plant species reported in the reviewed literature.

Crt. No.	Plant Species	Category of Species	Geographical Area	References
1	*Abies marocana* (Trab.) Emb. & Maire	Endangered endemic tree species (reported in the cited study; no specific legal or conservation framework indicated)	Marocco	Hatzilazarou et al., 2021 [43]
2	*Aconitum heterophyllum* Wall. ex Royle	Medicinal plant of conservation concern (reported in the cited study; no official framework specified)	India	Ahmad et al., 2023 [44]
3	*Aquilaria* sp.	Protected tree species of the tropical forest (protection framework reported in the cited study)	Malaysia	Adam et al., 2017 [45]
4	*Amburana cearensis* (Alemao) A.C.Sm.	Endangered tree species (reported in the cited study; no specific framework indicated)	Brazil	dos Santos et al., 2024 [46]
5	*Amphipterygium adstringens*	Endemic tree species heavily harvested for medicinal bark (conservation concern reported in the cited study)	Mexico	Beltrán-Rodríguez et al., 2022 [47]
6	*Andrographis paniculata* (Burm. f.) Nees	Endangered (reported in the cited study; no specific legal or conservation framework indicated)	India	Chamoli et al., 2014 [48]
7	*Anoectochilus roxburghii* (Wall.) Lindl.	Endangered (reported in the cited study; no specific legal or conservation framework indicated)	Vietnam	Ho et al., 2025 [49]
8	*Baccaurea courtallensis* (Wight) Muell. Arg.	Endangered (reported in the cited study; no specific legal or conservation framework indicated)	India	Sheba, 2019 [50]
9	*Blepharispermum subsessile* DC.	Threatened plant (included in a threatened plant list; specific framework not indicated)	India	Monalisa et al., 2024 [51]
10	*Boswellia serrata* Roxb. Ex Colebr	Threatened medicinal tree (reported in the cited study; no specific framework indicated)	India	Kumar and Tiwari, 2025 [52]
11	*Buchanania lanzan* Spreng.	Vulnerable (IUCN Red Data Book/IUCN Red List, as reported in the cited study)	India	Bhatnagar and Kumari, 2024 [53]
12	*Caesalpinia echinata* Lam.	Endangered with highly restricted population (reported in the cited study; no specific framework indicated)	Brazil	Cardoso et al., 1998 [54]
13	*Campomanesia phaea* (Berg) Landrum	Under risk of extinction (reported in the cited study; no specific framework indicated)	Brazil	Demétrio et al., 2021 [55]
14	*Camptotheca acuminata*	Rare, endemic, and endangered species (reported in the cited study; no specific framework indicated)	China	Wen and Yang, 2021 [56]
15	*Catamixis baccharoides* Thomson	Critically endangered (IUCN Red List, as reported in the cited study)	India	Dhiman et al., 2022 [57]
16	*Celtis toka* (Forssk.) Hepper & J.R.I.Wood	Critically endangered medicinal plant (reported in the cited study; no specific framework indicated)	Burkina Faso	Dabré et al., 2023 [58]
17	*Chamaecyparis obtusa* (Siebold & Zucc.) Endl.	Listed on the Red List of endangered species (Polish Red List, as reported in the cited study)	Poland	Górski et al., 2024 [59]
18	*Colchicum luteum* Baker	Rare and threatened medicinal plant (reported in the cited study; no specific framework indicated)	India	Rather et al., 2022 [60]
19	*Conradina glabra* Shinners	Endangered narrowly endemic scrub mint (reported in the cited study)	USA	Eserman et al., 2025 [61]
20	*Cornus officinalis* Torr. Ex Dur.	Endangered (reported in the cited study; no specific legal or conservation framework indicated)	China	Cao et al., 2016 [62]
21	*Coscinium fenestratum* (Gaertn.) Colebr.	Endangered (reported in the cited study; no specific legal or conservation framework indicated)	Inida	Danapur et al., 2020 [63]
22	*Cypripedium calceolus* L.	One of the most endangered orchid species in Europe (protected under European and Polish conservation legislation, as reported in the cited study)	Poland	Foremnik et al., 2021 [64]
23	*Daphne mezereum* L.	Protected under Polish national legislation	Poland	Nowakowska et al., 2023 [65]
24	*Decalepis arayalpathra* (Joseph & Chandra.) Venter	Endangered (reported in the cited study; no specific legal or conservation framework indicated)	Inida	Gangaprasad et al., 2005 [66]
25	*Euterpe edulis* Mart.	Endangered species of the Atlantic rainforest (reported in the cited study; no specific legal or conservation framework indicated)	Brazil	Cardoso et al., 2000 [67]
26	*Exacum bicolor* Roxb.	Endangered (reported in the cited study; no specific legal or conservation framework indicated)	India	Jeeshna and Paulsamy, 2011 [68]
27	*Gastrodia elata* Blume	Endangered (reported in the cited study; no specific legal or conservation framework indicated)	China	Chen et al., 2011 [69]
28	*Fritillaria roylei* D.Don	Critically endangered medicinal herb (reported in the cited study; no specific legal or conservation framework indicated)	India	Chandora et al., 2023 [70]
29	*Fritillaria ussuriensis* Maxim.	Endangered (reported in the cited study; no specific legal or conservation framework indicated)	China	Xie et al., 2024 [71]
30	*Froriepia subpinnata* (Ledeb.) Baill.	Endemic endangered medicinal plant (reported in the cited study; no specific legal or conservation framework indicated)	Iran	Jorkesh et al., 2020 [72]
31	*Hagenia abyssinica* Willd.	Endangered (reported in the cited study; no specific legal or conservation framework indicated)	Ethiopia	Assefa et al., 2010 [73]
32	*Harrisonia abyssinica* Oliv.	Endangered (reported in the cited study; no specific legal or conservation framework indicated)	Benin	Ogougbé et al., 2022 [74]
33	*Kirengeshoma palmata* Yatabe	Endangered (reported in the cited study; no specific legal or conservation framework indicated)	South Korea	Chang et al., 2007 [75]
34	*Ilex guayusa* Loes.	Endangered endemic Amazonian species (reported in the cited study; no specific legal or conservation framework indicated)	Ecuador	Carvalho et al., 2021 [76]
35	*Illicium griffithii* Hook.f. & Thomson	Endangered (IUCN Red List, as reported in the cited study)	India	Borah et al., 2021 [77]
36	*Juniperus drupacea* Labill.	Endangered in Greece (reported in the cited study; national conservation status)	Greece	Ioannidis et al., 2023 [78]
37	*Karomia gigas* (Faden) Verdc	Endangered tree species (reported in the cited study; no specific legal or conservation framework indicated)	Tanzania	Mapunda and Andrew, 2023 [79]
38	*Kelussia odoratissima* Mozaff.	Endangered medicinal species (reported in the cited study; no specific legal or conservation framework indicated)	Iran	Jahantab et al., 2022 [80]
39	*Khaya grandifoliola* C. Dc.	Endangered (reported in the cited study; no specific legal or conservation framework indicated)	Nigeria	Okere and Adegeye, 2011 [81]
40	*Lagerstroemia minuticarpa* Debberm. ex P.C. Kanjilal	Critically endangered tree species (reported in the cited study; no specific legal or conservation framework indicated)	India	Adhikari et al., 2019 [82]
41	*Lilium polyphyllum* D.Don ex Royle	Critically endangered (IUCN Red List, as reported in the cited study)	India	Dhyani et al., 2021 [83]
42	*Malania oleifera* Chun et S. Lee	Critically endangered (IUCN) Red List and Red Data Book of Chinese Plants, as reported in the cited study)	China	Liu et al., 2019 [84]
43	*Mirabilis himalaica* (Edgew.) Heimerl	Class I endangered medicinal plant (Chinese National Key Protected Wild Plants, as reported in the cited study)	China	Guo et al., 2024 [85]
44	*Oplopanax elatus* Miq.	Rare and endangered tree species (reported in the cited study; no specific legal or conservation framework indicated)	South Korea	Moon et al., 2006 [86]
45	*Origanum compactum* L.	Endemic medicinal plant (reported in the cited study; no specific legal or conservation framework indicated)	Morocco	Aboukhalid et al., 2017 [87]
46	*Oroxylum indicum* L. Kurz	Endangered (reported in the cited study; no specific legal or conservation framework indicated)	India	Samatha and Rama Swamy, 2020 [88]
47	*Panax quinquefolius* L.	Listed in Appendix II of CITES (Convention on International Trade in Endangered Species of Wild Fauna and Flora)	USA	Burkhart et al., 2012 [89]
48	*Paris polyphylla* Smith.	Endangered (reported in the cited study; no specific legal or conservation framework indicated)	India	Kumar et al., 2025 [90]
49	*Pausinystalia johimbe* (K. Schum)	Endangered (reported in the cited study; no specific legal or conservation framework indicated)	Cameroon	Tchoundjeu et al., 2004 [91]
50	*Phellodendron amurense* Rupr.	Endangered (reported in the cited study; no specific legal or conservation framework indicated)	China	Song and Zhang, 2017 [92]
51	*Phytolacca insularis* L.	Very rare plant (reported in the cited study; no specific legal or conservation framework indicated)	South Korea	Ahn and Lee, 2007 [93]
52	*Picrorhiza kurroa* Royle ex Benth	Endangered (reported in the cited study; no specific legal or conservation framework indicated)	India	Pandit et al., 2013 [94]
53	*Pilocarpus microphyllus* Stapf. ex Wardlew.	Threatened medicinal plant species (reported in the cited studies; no specific legal or conservation framework indicated)	Brazil	Monteiro et al., 2022; Amaral et al., 2022 [95,96]
54	*Pleodendron costaricense* N.Zamora, Hammel & Aguilar	Critically endangered tree (IUCN Red List, as reported in the cited study)	Costa Rica	Pillco Huarcaya et al., 2022 [97]
55	*Podophyllum hexandrum* (Royle) T.S.Ying	Endangered (reported in the cited study; no specific legal or conservation framework indicated)	India	Parasher et al., 2023 [98]
56	*Prunus ulmifolia* Franch.	Endangered species (reported in the cited study; no specific legal or conservation framework indicated)	Kazakhstan	Kirillov et al., 2025 [99]
57	*Psoralea corylifolia* L.	Rare medicinal plant (reported in the cited study; no specific legal or conservation framework indicated)	India	Jani et al., 2015 [100]
58	*Pterocarpus marsupium* Roxb.	Endangered tree species (reported in the cited study; no specific legal or conservation framework indicated)	India	Ahmad et al., 2022 [101]
59	*Pterocarpus santalinus* L.	Endemic and endangered species (reported in the cited study; no specific legal or conservation framework indicated)	India	Babar et al., 2012 [102]
60	*Salvia majdae* (Rech.f. & Wendelbo) Sytsma	Endemic medicinal plant (reported in the cited study; no specific legal or conservation framework indicated)	Iran	Ajani et al., 2022 [103]
61	*Saraca asoca* (Roxb.) W.J.de Wilde	Vulnerable (IUCN Red List) and endangered (Conservation Assessment and Management Plan, CAMP), as reported in the cited study	India	Bhat et al., 2024 [104]
62	*Saussurea costus* (Falc.) Lipschitz	Endangered (reported in the cited study; no specific legal or conservation framework indicated)	India	Pandey et al., 2007 [105]
63	*Saussurea involucrate* DC.	Endangered (reported in the cited study; no specific legal or conservation framework indicated)	Mongolia	Dashzeveg et al., 2017 [106]
64	*Silybum marianum* L.	Endangered (reported in the cited study; no specific legal or conservation framework indicated)	Iran	Hojati et al., 2025 [107]
65	*Sinopodophyllum emodi* (Royle) T.S.Ying	Endangered (reported in the cited study; no specific legal or conservation framework indicated)	China	Zhao et al., 2011 [108]
66	*Sorbus caloneura* L.	Endangered (reported in the cited study; no specific legal or conservation framework indicated)	China	Guo et al., 2023 [109]
67	*Soymida febrifuga* A. Juss.	Endangered (reported in the cited study; no specific legal or conservation framework indicated)	India	Hetalba et al., 2025 [110]
68	*Strychnos potatorum* L.f.	Endangered forest tree species (reported in the cited study; no specific legal or conservation framework indicated)	India	Kagithoju et al., 2013 [111]
69	*Swertia chirayita* (Roxb. ex Fleming) H. Karst.	Critically endangered medicinal herb (reported in the cited study; no specific legal or conservation framework indicated)	India	Pradhan and Badola, 2010 [112]
70	*Taxus baccata* L.	Endangered tree (reported in the cited study; no specific legal or conservation framework indicated)	Iran	Hematzadeh et al., 2023 [113]
71	*Taxus wallichiana* Zucc	Endangered (IUCN Red List, as reported in the cited study)	India	Dissanayake et al., 2023 [114]
72	*Ternstroemia cameroonensis* sp. nov.	Critically endangered (IUCN Red List Categories and Criteria, version 3.1, 2012), as reported in the cited study	Cameroon	Cheek et al., 2017 [115]
73	*Trillium govanianum* Wall. ex D. Don	Endangered traditional medicinal herb (reported in the cited study; no specific legal or conservation framework indicated)	India	Chauhan et al., 2018 [116]
74	*Vepris onanae* (Aubrév. & Pellegr.)	Critically endangered (IUCN Red List Categories and Criteria, version 3.1, 2012), as reported in the cited study	Cameroon	Cheek et al., 2022 [117]
75	*Warburgia salutaris* (Bertol.f.) Chiov.	Endangered (reported in the cited study; no specific legal or conservation framework indicated)	Kenya	Kioko et al., 1999 [118]
76	*Zanthoxylum armatum* DC.	Endangered (reported in the cited study; no specific legal or conservation framework indicated)	India	Purohit et al., 2017 [119]
77	*Zygophyllum potaninii* Maxim.	Endangered species with regional conservation status (reported in the cited study)	Mongolia	Bayarmaa et al., 2018 [120]

Abbreviations: IUCN, International Union for Conservation of Nature; CITES, Convention on International Trade in Endangered Species of Wild Fauna and Flora; CAMP, Conservation Assessment and Management Plan. Where no official conservation framework was identified in the cited publication, the conservation status corresponds to the designation reported by the original study.

## Data Availability

No new data were created or analyzed in this study. Data sharing is not applicable to this article.
